# Disrupted cardiac fibroblast BCAA catabolism contributes to diabetic cardiomyopathy via a periostin/NAP1L2/SIRT3 axis

**DOI:** 10.1186/s11658-023-00510-4

**Published:** 2023-11-22

**Authors:** Qing-Bo Lu, Xiao Fu, Yao Liu, Zi-Chao Wang, Shi-Yi Liu, Yu-Chao Li, Hai-Jian Sun

**Affiliations:** 1 Department of Basic Medicine, Wuxi School of Medicine, Jiangnan University, Wuxi, 214122 China; 2 Department of Endocrine, Affiliated Hospital of Jiangnan University, Jiangnan University, Wuxi, 214125 China; 3 Department of Cardiac Ultrasound, The Fourth Affiliated Hospital of Nanjing Medical University, Nanjing, 210000 Jiangsu China; 4 State Key Laboratory of Natural Medicines, China Pharmaceutical University, No. 24 Tongjia Lane, Nanjing, 210009 China

**Keywords:** Periostin, Diabetes, Cardiomyopathy, BCAA catabolism, Glucosyringic acid

## Abstract

**Background:**

Periostin is an extracellular matrix protein that plays a critical role in cell fate determination and tissue remodeling, but the underlying role and mechanism of periostin in diabetic cardiomyopathy (DCM) are far from clear. Thus, we aimed to clarify the mechanistic participation of periostin in DCM.

**Methods:**

The expression of periostin was examined in DCM patients, diabetic mice and high glucose (HG)-exposed cardiac fibroblasts (CF). Gain- and loss-of-function experiments assessed the potential role of periostin in DCM pathogenesis. RNA sequencing was used to investigate the underlying mechanisms of periostin in DCM.

**Results:**

A mouse cytokine antibody array showed that the protein expression of periostin was most significantly upregulated in diabetic mouse heart, and this increase was also observed in patients with DCM or HG-incubated CF. Periostin-deficient mice were protected from diabetes-induced cardiac dysfunction and myocardial damage, while overexpression of periostin held the opposite effects. Hyperglycemia stimulated the expression of periostin in a TGF-β/Smad-dependent manner. RNA sequencing results showed that periostin upregulated the expression of nucleosome assembly protein 1-like 2 (NAP1L2) which recruited SIRT3 to deacetylate H3K27ac on the promoters of the branched-chain amino acid (BCAA) catabolism-related enzymes BCAT2 and PP2Cm, resulting in BCAA catabolism impairment. Additionally, CF-derived periostin induced hypertrophy, oxidative injury and inflammation in primary cardiomyocytes. Finally, we identified that glucosyringic acid (GA) specifically targeted and inhibited periostin to ameliorate DCM.

**Conclusion:**

Overall, manipulating periostin expression may function as a promising strategy in the treatment of DCM.

**Supplementary Information:**

The online version contains supplementary material available at 10.1186/s11658-023-00510-4.

## Background

The prevalence of diabetes is growing rapidly, and diabetic complications are becoming a global public health problem that has troubled hundreds of millions of people worldwide [1, 2]. Amongst all the diabetic complications, cardiovascular disorders are recognized as the major causes of disability and mortality rate of diabetic individuals [3]. Diabetic cardiomyopathy (DCM) is primarily manifested by cardiac hypertrophy, fibrosis, apoptosis, and insufficiency, eventually leading to heart failure and sudden death in diabetic populations [4]. Compelling evidence has demonstrated that impaired cardiac insulin metabolic signaling, oxidative stress, elevations in advanced glycation end products, mitochondrial dysfunction, endoplasmic reticulum stress, inflammatory response, microvascular dysfunction, abnormalities in O-linked N-acetylglucosamine and exosomal pathways have been linked to the pathologies of DCM [4–8]. Despite growing advances in the preclinical and clinical studies during the last decades, the pathophysiology of DCM remains to be fully elucidated. To date, although a panel of clinically effective drugs have shown promise in restoring cardiac function in experimental DCM models, their clinical efficacy is not sufficient for the management of DCM [9]. Therefore, there is an urgent unmet medical need to develop efficient therapeutic strategies that can delay or ameliorate DCM.

Periostin is a secreted matricellular protein predominantly expressed in various tissues, including cardiovascular system [10, 11]. Secreted protein is composed of four fascicular protein domains that are homologous to insect protein fascicular protein I, which is involved in cell–matrix crosstalk [12]. A number of studies underscore the important role of periostin in extracellular matrix (ECM) development and maturation, as well as cellular adhesion [13]. Accumulating evidence underpins the putative role of periostin in the development of several cardiovascular diseases, including cardiac fibrosis [14, 15], atrial fibrillation [16], aortic dissection [17], acute myocardial infarction [18], arterial calcification [19], atherosclerosis [20], and pulmonary arterial hypertension [21, 22]. Highly expressed periostin is associated with fibroproliferative diseases in the heart, which may be related to the activation of transforming growth factor-β (TGF-β)/bone morphogenetic protein (BMP) signaling [12]. Deletion of periostin-positive myofibroblasts reduces is capable of reducing collagen production and scar formation after myocardial infarction, indicating that periostin-expressing cell type is necessary for and fibrosis in the heart [23]. Gibb et al. have demonstrated that targeting the signaling pathways responsible for myofibroblast formation and persistence by specifically downregulating the periostin-specific fibroblast population is sufficient to reverse tissue fibrosis and cardiac dysfunction in a mouse model of pressure-overload [24]. This finding further confirms the ability of periostin-expressing fibroblasts to induce fibrosis and cardiac dysfunction in heart failure [24]. These findings might shed light on the potential applications of periostin-specific therapies for cardiovascular diseases. A network analysis revealed that periostin levels are strongly clustered with C-reactive protein and interleukin-6 in diabetic patients [25]. In this study, our cytokine antibody microarray results showed that periostin was among the most upregulated expressed genes in the heart of DCM mice when compared with those from control mice. Similar results were also observed in patients suffering from DCM. It is highly probable that periostin may be an important mediator in the pathogenesis of DCM. However, whether periostin mediates diabetes-induced cardiac damage and its role in the etiologies of DCM are unclear. In the present study, genetic and pharmacological regulations of periostin were used to explore the exact role and mechanism of periostin in DCM pathogenesis.

Our current study found increased expression of periostin in diabetic mouse heart, high glucose (HG)-exposed cardiac fibroblasts (CF), along with increased serum periostin levels in patients with DCM. Deletion of periostin attenuated, while overexpression of periostin worsened diabetes-related cardiac dysfunction and injury. Mechanistically, periostin upregulated nucleosome assembly protein 1-like 2 (NAP1L2) to recruit SIRT3 to deacetylate H3K27ac on the promoters of the branched-chain amino acids (BCAAs) catabolism-related enzymes BCAT2 and PP2CM, leading to overproduction of BCAAs in CF and subsequent cardiac fibrosis. CF-secreted periostin paracrinically induced cardiomyocyte hypertrophy and oxidative damage. We further screened that a phytochemical called glucosyringic acid (GA) interacted with and suppressed periostin expression, thereby ameliorating the symptoms of DCM in mice. Our results provided potential mechanistic insight into the role of periostin in the pathophysiology of DCM, and suggested that GA served as a promising compound for therapeutic interventions against DCM by targeting periostin.

## Methods

### Reagents and chemicals

The information for primary and second antibodies (Additional file 1: Table S1), primers (Additional file 1: Table S2, S3), and natural product library (Additional file 1: Table S4) were listed in Additional file 1. Extended detailed methods are presented in Additional file 2.

### Animals

Male C57BL/6 J mice aged 7–8 weeks were purchased from Experimental Animal Center of Yangzhou University. High-fat diet (HFD, 60% fat) was procured from FBSH Biotechnology Co., Ltd. (Shanghai, China). Periostin-deficient mice were purchased from the Jackson Laboratory (Bar Harbor, ME, USA) as previously described [22]_._ All mice were housed in pathogen-free cages, with free access to autoclaved food and reverse-osmosis water, and the animals were caged under a controlled temperature and humidity room on a 12-h light/dark cycle. HFD feeding and STZ injection were used to induce type 2 diabetic mice as previously depicted [26, 27]. In brief, mice were fed with a HFD for 8 weeks and then subjected to an intraperitoneal injection of STZ (formulated in 0.1 M citrate buffer, pH 4.5, 120 mg/kg). The control mice were fed with a normal diet for 8 weeks and received a single injection of the same volume of sodium citrate buffer. Five days after STZ injection, mice with hyperglycemia (6-h fasting blood glucose more than 11.1 mM) were indicative of diabetes. To specifically overexpress periostin in murine hearts, mice were intravenously subjected to a single injection of AAV9 vectors encoding periostin or negative AAV9 vectors (10^11^ viral genome particles for each mouse) under the control of fibroblast-specific protein 1 (FSP1) promoters one week after the first injection of STZ [28]. Similarly, AAV9 vectors carrying a FSP1 promoter that control shRNA or periostin shRNA (10^11^ viral genome particles for each mouse) were intravenously introduced into each mouse to specifically downregulate the myocardial fibroblasts-located periostin in murine hearts. After six weeks, transthoracic echocardiography in each mouse anesthetized with 0.8% isoflurane were conducted by using Vevo 2100 high-resolution imaging system equipped with a 30-MHz probe (VisualSonics, Toronto, ON, Canada) on a heating pad. In addition, the potential role of glucosyringic acid (GA) in DCM was also determined. Briefly, one week after the first injection of STZ, the diabetic mice were treated with different doses of GA (10, 20, 40 mg) every other day until for the subsequent 6 weeks. After that, the cardiac function was assessed. All procedures were conducted by the same operator. The systolic function and diastolic function was calculated using pulsed-wave Doppler imaging as we previously described [29]. For euthanasia, animals were anesthetized with 5% isoflurane, anaesthesia was confirmed via tail pinch, and then sacrificed by cervical dislocation before cardiac tissue removal. All procedures for animal studies were reviewed and approved by the Ethics Committee of China Pharmaceutical University (approval number: 202101016). The experiments were complied with the Care and Use of Laboratory Animals published by the US National Institutes of Health (NIH Publication, revised 2011), the Basel Declaration, and the ethical standards in the 1964 Declaration of Helsinki.

### Serum collection from participants

All human serum samples were obtained with the informed consent of the patients and the protocols were reviewed and approved by the Ethics Committee for the Use of Human Subjects of the Nanjing Medical University (approval number: 20180705-K048), and the experiments involving humans were also in accordance with the Declaration of Helsinki. In this study, 35 healthy participants, 36 diabetic patients with normal cardiac function, and 33 diabetic patients having symptoms and signs of heart failure were enrolled from The Fourth Affiliated Hospital of Nanjing Medical University in compliance with the previous diagnose of DCM in patients [1]. All the participants read and signed an informed consent form. The information for the enrolled patients was provided in Additional file 1: Table S5. Serum samples of each participate were collected in the morning after an overnight fasting period. Samples were centrifuged and subsequently aliquoted and stored at − 80 °C prior to analysis.

### Cell culture

Primary cardiac myocytes, cardiac endothelial cells and fibroblasts were isolated and cultured from the neonatal Sprague Dawley rats purchased from Experimental Animal Center of Yangzhou University as we previously described [29]. When primary CF populations reached 50–60% confluence, they were challenged by either normal glucose (NG) or high glucose (HG) culture medium. The NG medium was a mixture of 5.5 mM of glucose with 27.8 mM of mannitol. HG culture medium was obtained using the DMEM complete medium (5.5 mM glucose) supplemented with D-glucose to a final concentration of 33.3 mM [30]. The primary CF were transfected with lentivirus-mediated periostin shRNA or periostin plasmid prior to stimulation with different concentrations of glucose in CF. After that, the cells were harvested for subsequent experiments. To determine the function of CF-derived periostin in cardiomyocyte behaviors, we transfected CF with either empty vectors or periostin overexpression plasmids. The conditioned medium was prepared from CF after 48 h of transfection. After that, the conditioned medium was added to the cardiomyocytes for 48 h, the cardiomyocyte hypertrophy and apoptosis were then determined. All experiments were carried out at least three separate stably transfected populations. Human embryonic kidney (HEK) 293 T cells were obtained from Huzhen Biotechnology Co., Ltd. (Shanghai, China) and cultured in DMEM supplemented with 10% foetal bovine serum (FBS) and 1% penicillin–streptomycin in an incubator of 5% CO_2_ at 37 °C. The cell viability was examined by a Cell Counting Kit-8 kit and EdU incorporation as we previously described [31].

### Histological examination

The cardiac tissues were fixed in 4% paraformaldehyde, embedded in paraffin, and then sliced into 5 μm thick sections. The sections were stained with hematoxylin and eosin (H&E), and Sirius red. The morphological changes of the cardiac tissues were photographed by an optical microscope (Olympus, Tokyo, Japan), and the cardiomyocyte area was calculated from more than 100 cells in each cardiac section using ImageJ quantitative analysis system (Image Pro-Plus version 6.0). The collagen content of were observed in six fields from each imaged section and the collagen volume fraction was quantified.

### Chromatin immunoprecipitation (ChIP) assay

After the required treatment, the cells were harvested to conduct ChIP experiment to show the DNA sequences of periostin promoters bound to Smad2 or Smad3. In accordance with the manufacturer’s constructions, experiments were carried out using the ChIP kit (Ab185913, Abcam, Cambridge, UK). The cells were crosslinked with 1% formaldehyde for 10 min, and stopped with 125 mM glycine. After chromosomal separation, the sheared DNA fragments associated with the binding proteins were collected. The aliquots of lysates in each chromatin solution were subjected to immunoprecipitation with Smad2 antibody, Smad3 antibody, or pre-immune IgG (CST, catalog no. 2729, 1:200) overnight at 4 °C, and the complex containing targeted proteins and its binding DNAs were isolated. The DNA was then released by reverse cross-linking, and the protein was digested. RT-qPCR was performed to detect the precipitated genomic DNA with primers specific for the Smad2 or Smad3 binding site within periostin promoters. Non-precipitated genomic DNA input was amplified as an input control. The ChIP of H3K27ac (Abcam) cells were lysed in lysis-buffer and chromatin was sonicated to a fragment size of 150 to 300 bp. The enrichment of H3K27ac within the promoters of BCAT2, PP2Cm, Col I and α-SMA was measured by RT-qPCR. The ChIP primers are listed in Additional file 1: Table S6. The normalized enrichment value was expressed as the subtraction of the IP relative value with the input relative value.

### Statistical analysis

In this study, the cellular and molecular experiments were independently repeated for at least 3 times. The animal assays involved were independently repeated for at least 6 mice. Data from replications were averaged and expressed as mean value ± standard deviation (SD). The statistical analysis was conducted by GraphPad Prism 5.0 (GraphPad Software, Inc., San Diego, CA, USA). Unpaired *t*-test was utilized to determine differences between two groups. The statistical significance of correlations was assessed by Pearson’s correlation coefficient analysis. Analysis of variance (ANOVA) was performed for the comparison of multiple groups. Bonferroni post-hoc testing was used following ANOVA for analyzing all comparisons among groups. *P* < 0.05 was deemed as statistically significant.

## Results

### Highly expressed periostin in diabetic mouse heart, HG-induced CF and patients with DCM

**Fig. 1:**
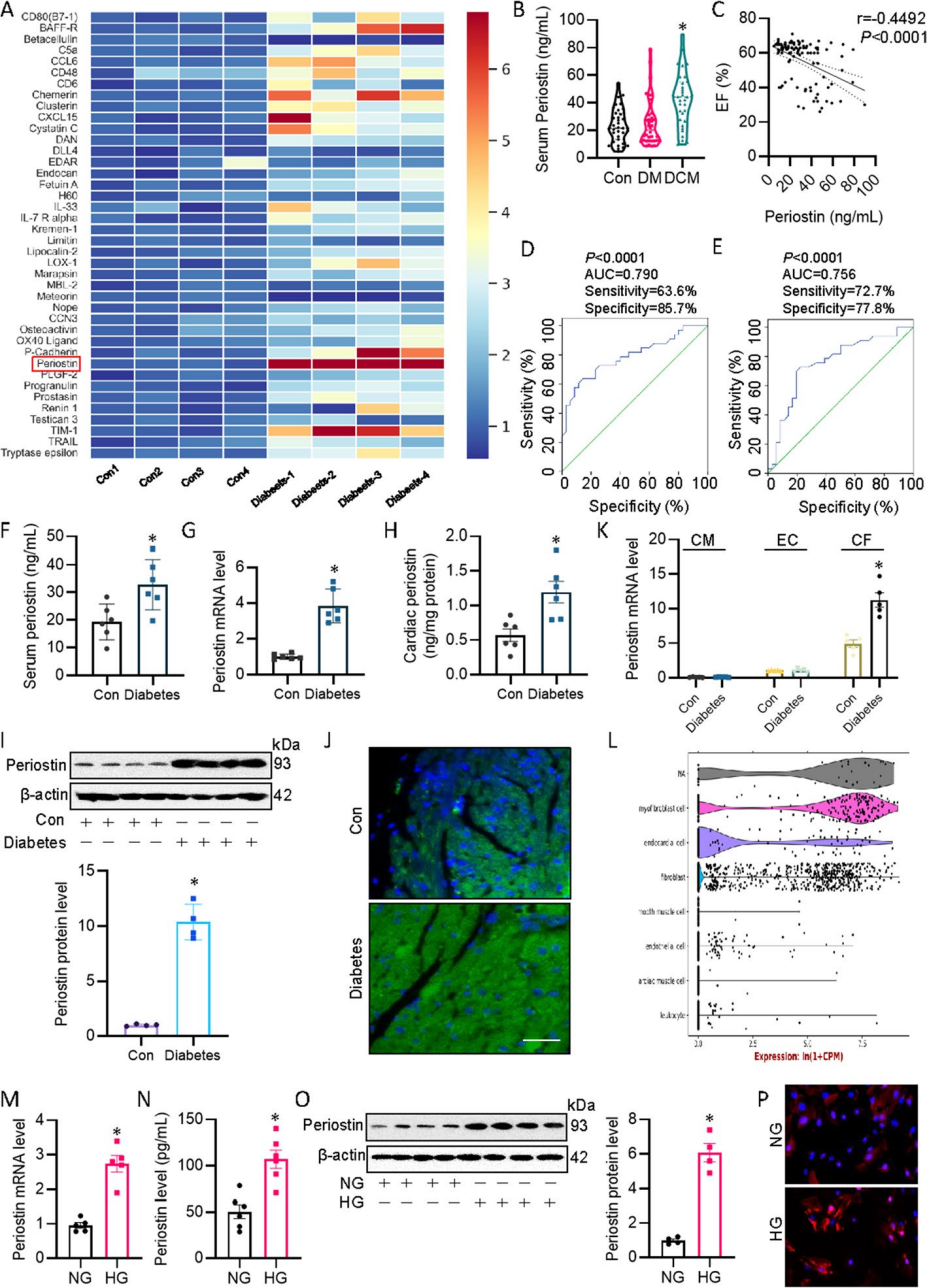
Expression of periostin in patients with DCM, diabetic mice and HG-exposed CF. **A** A mouse cytokine antibody array showing the cytokine protein changes in heart tissues from control or diabetic mice. **B** Serum periostin levels in healthy participates, diabetic patients with normal cardiac function, and diabetic patients with heart failure. **C** Negative correlations between periostin levels and EF. **D** ROC curve for the diagnostic performance of serum priostin between healthy participants and DCM patients. **E** ROC curve for the diagnostic performance of serum priostin between diabetic patients with normal cardiac function and DCM patients. **F** Serum periostin levels in control and diabetic mice. **G** Relative mRNA level of periostin in the hearts from control and diabetic mice. **H** Cardiac periostin levels in control and diabetic mice using ELISA. **I** Representative blots and quantitation of periostin protein in control and diabetic mice. **J** Immunofluorescence showing the periostin expression in control and diabetic mice. **K** Cardiac periostin mRNA level in isolated cardiomyocytes (CM), cardiac endothelial cells (EC), and cardiac fibroblasts (CF) from control and diabetic mice. **L** Tabula Muris database showing the distribution of periostin different cells within the hearts. **M** Relative mRNA level of periostin in CF exposed to NG or HG. **N** The protein level of periostin in CF exposed to NG or HG using ELISA. **O** The protein level of periostin in CF exposed to NG or HG using western blot. **P** Immunofluorescence showing the periostin expression in CF exposed to NG or HG. *n* = 4–6. *P < 0.05 versus Control (Con) or normal glucose (NG). Differences between groups were assessed with ANOVA followed by Bonferroni post-hoc test (**B**). The statistical significance of correlations was assessed by Pearson’s correlation coefficient analysis (**C**). The *P*-value was calculated by unpaired two-tailed Student’s *t*-test (**F**-**K**)

Firstly, we obtained a mouse cytokine antibody array to probe the potential therapeutic targets in DCM. The protein profiling assay with this cytokine antibody array showed the levels of 32 proteins were higher in diabetic hearts when compared with those in normal heart. Of these, the protein expression of periostin was increased by 14.3 fold in diabetic hearts, making it the top one upregulated protein in DCM (Fig. 1A). This drove us to explore the clinical relevance of periostin in DCM. Compared with healthy controls, serum levels of periostin were significantly enhanced in patients suffering from DCM (Fig. 1B), and serum periostin levels were negatively correlated with ejection fraction in enrolled participants (Fig. 1C). Importantly, the analysis of receiver-operating characteristic (ROC) curve revealed that the changes in serum periostin levels might serve as a possible biomarker for cardiomyopathy in diabetic patients (Fig. 1D, E). In consistence with clinical data, serum levels of periostin were also raised in DCM mice (Fig. 1F). RT-PCR, ELISA, immunoblotting and immunofluorescence consistently revealed higher periostin level in heart tissues of diabetic mice (Fig. 1G–J). Intriguingly, chronic hypercemia upregulated periostin transcription level in cultured cardiac fibroblasts (CF) from diabetic mice, without changing periostin mRNA level in isolated cardiomyocytes (CM) and cardiac endothelial cells (EC) from diabetic mice (Fig. 1K). To establish which cardiac cell type contributed to the upregulated expression levels of periostin, we investigated the expression profiles of periostin in mouse heart with the aid of the Tabula Muris database. Single cell sequencing of mouse heart tissues showed that periostin was mainly expressed in CF among the top 5 mouse heart cell types (Fig. 1L). In parallel, the mRNA and protein levels of periostin were significantly elevated in primary CF upon exposure of high glucose (HG) (Fig. 1M–P). As periostin is a prototypical myofibroblast marker, it is interesting to know whether TGF-β1 or angiotensin II had an impact on the expression of periostin in CF. Results showed that transforming growth factor β1 (TGF-β1) had the strongest ability to upregulate the mRNA level of periostin in CF, followed by HG and angiotensin II (Ang II) (Additional file 2: Fig. S1). These results support the close relationship between cardiac periostin levels and DCM.

### Periostin deficiency attenuates cardiac injury and dysfunction in diabetic mice

**Fig. 2:**
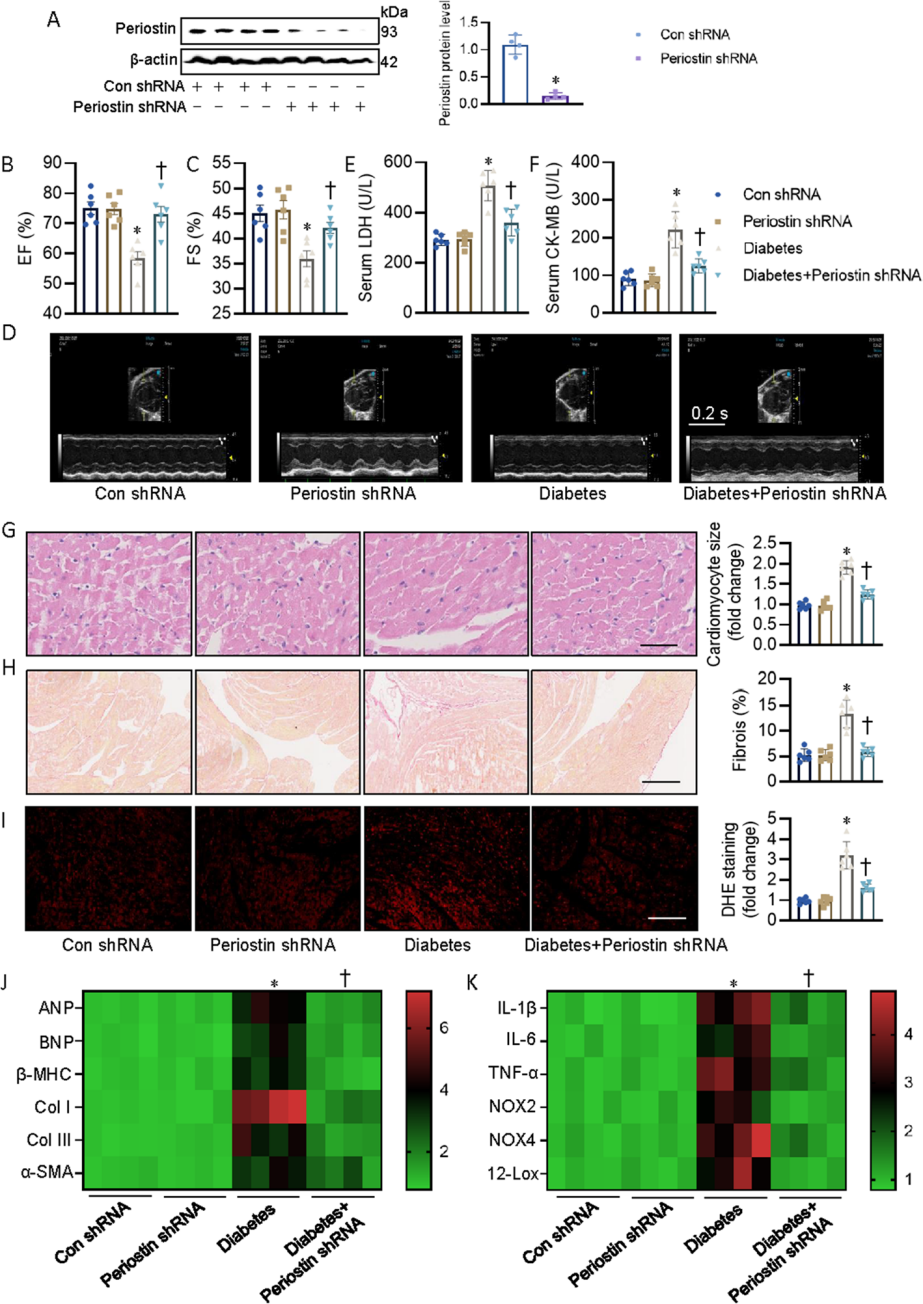
Periostin-deficient mice were resistant to diabetes-indcued cardiac dysfunction. **A** Representative blots and quantitation of periostin protein. **B**, **C** Left ventricle EF and FS were quantified. **D** Representative echocardiographic images showing the effects of perisotin knockdown on cardiac function in control and diabetic mice. **E** Serum LDH levels in mice. **F** Serum CK-MB levels in mice. **G** Representative photographs of the myocardium with H&E staining (Scale bar = 100 μm). **H** Representative photographs of the myocardium with Sirus red staining (Scale bar = 100 μm). **I** Representative images of the myocardium with DHE staining (Scale bar = 200 μm). **J** Heatmap showing the mRNA levels of pro-hypertrophic genes (*ANP*, *BNP*, *β-MHC*) and pro-fibrogenic genes (*Col I*, *Col III*, *α-SMA*). **K** Heatmap showing the mRNA levels of pro-inflammtory genes (*IL-1β*, *IL-6*, *TNF-α*) and pro-oxidative genes (*NOX2*, *NOX4*, *12-LOX*). *n* = 4–6. *P < 0.05 versus Control (Con), †*P* < 0.05 versus Diabetes. Differences between groups were assessed with ANOVA followed by Bonferroni post-hoc test

Next, we evaluated the effect of high-fat diet (HFD) plus low dose of STZ on the biometric, morphological, and echocardiographic characteristics in both control and periostin shRNA mice. Consumption of a HFD intake and STZ injection resulted in elevated fasting blood glucose (FBG), enhanced homeostatic model assessment for insulin resistance (HOMA-IR) index, and increased serum cholesterol and triacylglycerols in both control and periostin shRNA mice, effects that were similar in all mouse groups (Additional file 1: Table S7). Noninvasive transthoracic echocardiography disclosed that ablation of periostin led to a significant improvement of diabetes-elicited cardiac diastolic and systolic dysfunction (Fig. 2A–D, Additional file 1: Table S7). Consistent with the improvement of cardiac function, serum levels of lactate dehydrogenase (LDH) and creatine kinase-cardiac (CK-MB) were lower in periostin-deficient mice response to HFD and STZ (Fig. 2E, F), suggesting that periostin knockout mice were resistant to diabetes-induced heart damage. Under normal conditions, no significant difference was found in cardiac injury between control and periostin shRNA mice (Fig. 2G–I). However, HFD and STZ promoted cardiac hypertrophy, fibrosis and oxidative stress in control mice only, but not in the periostin-deficient mice, as evidenced by H&E staining, sirius red staining and DHE staining (Fig. 2G–I). Likewise, loss of periostin suppressed diabetes-triggered upregulations of the pro-hypertrophic genes (*ANP*, *BNP*, *β-MHC*), pro-fibrogenic genes (*Col I*, *Col III*, *α-SMA*), pro-inflammtory genes (*IL-1β*, *IL-6*, *TNF-α*) and pro-oxidative genes (*NOX2*, *NOX4*, *12-LOX*) at the mRNA levels (Fig. 2J, K).

### Periostin overexpression exacerbated diabetes-induced cardiomyopathy

**Fig. 3:**
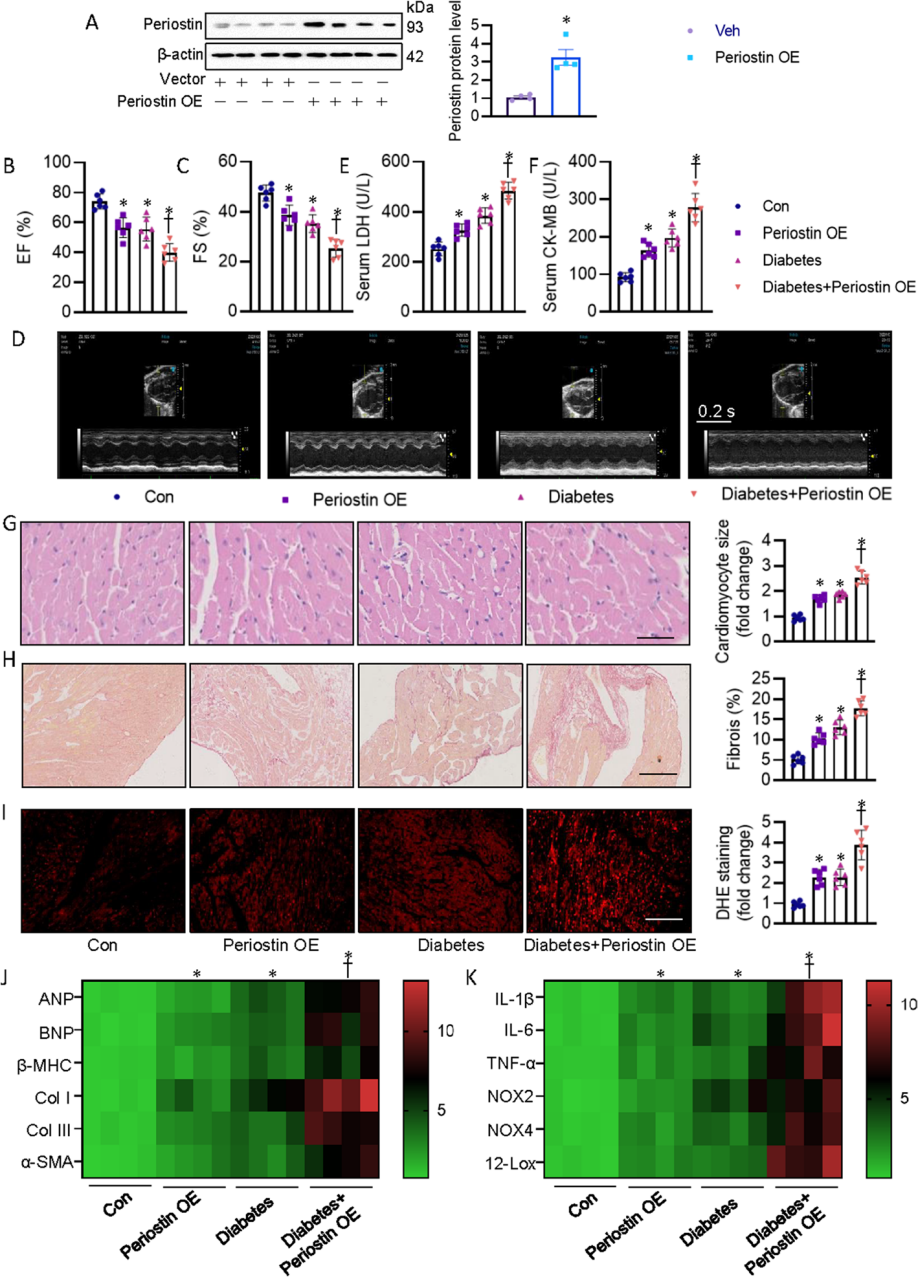
Periostin overexpression worsened cardiac injury and dysfunction in diabetic mice. **A** Representative blots and quantitation of periostin protein. **B**, **C** Left ventricle EF and FS were quantified. **D** Representative echocardiographic images showing the effects of perisotin knockdown on cardiac function in control and diabetic mice. **E** Serum LDH levels in mice. **F** Serum CK-MB levels in mice. **G** Representative photographs of the myocardium with H&E staining (Scale bar = 100 μm). **H** Representative photographs of the myocardium with Sirus red staining (Scale bar = 100 μm). **I** Representative images of the myocardium with DHE staining (Scale bar = 200 μm). **J** Heatmap showing the mRNA levels of pro-hypertrophic genes (*ANP*, *BNP*, *β-MHC*) and pro-fibrogenic genes (*Col I*, *Col III*, *α-SMA*). **K** Heatmap showing the mRNA levels of pro-inflammtory genes (*IL-1β*, *IL-6*, *TNF-α*) and pro-oxidative genes (*NOX2*, *NOX4*, *12-LOX*). *n* = 4–6. *P < 0.05 versus Control (Con), †*P* < 0.05 versus Diabetes. Differences between groups were assessed with ANOVA followed by Bonferroni post-hoc test

To further explore the role of periostin in DCM, diabetic hearts were infected with AAV vectors encoding mouse periostin. As expected, AAV-mediated periostin overexpression significantly elevated the periostin protein levels in heart tissues (Fig. 3A). Although periostin overexpression itself did not exert any effect on body, nor did it affected global metabolic profiles (Additional file 1: Table S8), its ectopic expression induced cardiac dysfunction, and aggravated diabetes-evoked changes in echocardiographic indices (Fig. 3B–D, Additional file 1: Table S8). Consistent with these results, overexpression of periostin caused increases in serum markers of cellular damage, LDH and CK-MB, with a more pronounced effect under diabetic state (Fig. 3E, F). Histological analysis by H&E staining, sirius red staining and DHE staining also showed that upregulation of periostin accelerated cardiomyocyte hypertrophy, fibrosis and oxidative injury in STZ/HFD-induced diabetic mice as compared with control mice (Fig. 3G–I). Transcriptional analysis of heart revealed that cardiac hypertrophy, fibrosis, inflammation and oxidative burst were intensified in diabetic mice with periostin overexpression (Fig. 3J, K). These findings indicated that periostin might be a driving factor for the development of DCM.

### Periostin knockdown blunted, while periostin overexpression aggravated HG-induced CF activation

**Fig. 4:**
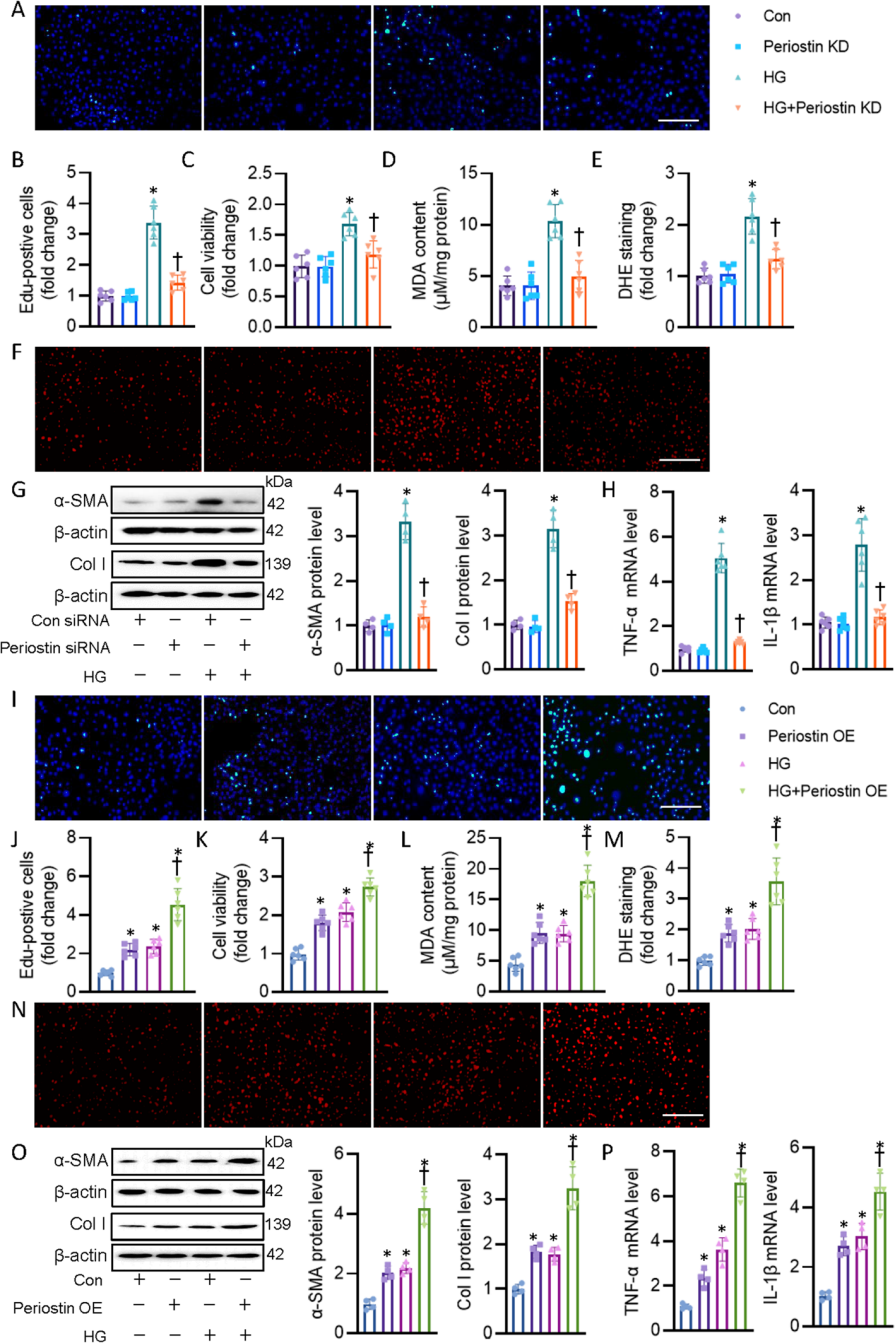
Periostin knockdown attenuated, while periostin overexpression intensified HG-induced CF activation. **A**, **B** Representative photographs and quantitative analysis of EdU-positive cells. **C** Cell viability. **D** MDA contents. **E**, **F** Representative photographs and quantitative analysis of DHE staining. **G** Representative blots and quantitation of α-SMA and collagen I. **H** Representative mRNA levels of TNF-α and IL-1β. **I**, **J** Representative photographs and quantitative analysis of EdU-positive cells. **K** Cell viability. **L** MDA contents. **M**, **N** Representative photographs and quantitative analysis of DHE staining. **O** Representative blots and quantitation of α-SMA and collagen I. **P** Representative mRNA levels of TNF-α and IL-1β. *n* = 4–6. *P < 0.05 versus Control (Con), †*P* < 0.05 versus HG. Differences between groups were assessed with ANOVA followed by Bonferroni post-hoc test

To further ascertain the protective role of periostin against DCM, we next determined the effect of periostin knockdown and overexpression on CF activation. As shown in Fig. 4A–C and Additional file 2: Fig. S2A, silencing periostin mitigated HG-elicited CF proliferation, as demonstrated by EdU staining and CCK-8 assay. HG stimulation significantly promoted the formation of MDA and superoxide anions in CF, while this was normalized by depletion of periostin (Fig. 4D–F). Immunoblotting assay revealed lower protein expressions of α-SMA and Col I in periostin-deficient CF when compared with that in vehicle-treated cells (Fig. 4G), indicating that periostin might affect myofibroblast transformation and subsequent cardiac fibrosis. In addition, HG environment lifted the mRNA levels of *IL-1β* and *TNF-α* in CF, whereas this inflammatory phenotype was not observed in periostin-deficient cells (Fig. 4H). In contrast to periostin knockdown studies, the proliferation, oxidative burst, inflammation and myofibroblast transformation of CF were further deteriorated in response to HG after overexpression of periostin (Fig. 4I–P, Additional file 2: Fig. S2B). Although hyperglycemia did not change the level of periostin in cardiomyocytes, global knockout of periostin can inhibit cardiomyocyte hypertrophy caused by diabetes. This drives us to investigate whether CF-released periostin could affect cardiomyocyte functions. CF-derived periostin level could be detectable in CF under NG condition, whereas this secreted protein was significantly enhanced in response to HG (Additional file 2: Fig. S3A). Thus, we further studied whether CF-secreted periostin played a role in cardiomyocyte behaviors by transferring the conditioned medium from CF to CM. The conditioned medium containing periostin from CF inhibited the CM viability and caused cellular LDH release in CM (Additional file 2: Fig. S3B-D). The conditioned medium containing periostin from CF resulted in CM hypertrophy, along with higher mRNA levels of pro-hypertrophic genes, including *ANP*, *BNP*, *β-MHC* (Additional file 2: Fig. S3E, F)*.* Additionally, TUNEL staining showed that CF-derived periostin enhanced CM apoptosis (Additional file 2: Fig. S3G), accompanied by the upregulation of pro-apoptotic gene Bax and downregulation of anti-apoptotic gene Bcl-2 (Additional file 2: Fig. S3H).

### Periostin is induced by HG in a TGF-β/Smad dependent manner

We further identified the potential mechanisms that underlie HG-induced overexpression of periostin in CF. The luciferase activity from a series of deletion mutants of periostin promoter constructs were measured to discern the potential promoter regions that contributing to periostin upregulation in HG-exposed CF. The luciferase activity in a full-length promoter region of periostin tended to be higher in CF after treatment with HG (Additional file 2: Fig. S4A, B). Nevertheless, the luciferase activity was not further increased when a small region (− 1495 to − 1195) of periostin promoter was deleted under HG conditions (Additional file 2: Fig. S4A, B), suggesting that this promoter region (− 1495 to − 1195) might mediate the upregulation of periostin transcription levels. Previously, the protein expression of periostin was reported to be upregulated in neu-positive breast cancer cells through the PI3K/Akt signaling [32]. TGF-β acts on the receptor-activated Smad2 and Smad3 to regulate homeostatic functions of fibroblasts by regulating the expression of periostin [33, 34]. Therefore, we investigated whether HG upregulated the transcriptional and translational levels of perisotin by regulating the PI3K/Akt and/or TGF-β/Smad pathways. Western blots and RT-PCR results showed that induction of perisotin by HG in CF was suppressed by TGF-β neutralizing antibody, Smad2/3 knockdown (Additional file 2: Fig. S2C, D), rather than PI3K or Akt inhibitors (Additional file 2: Fig. S4C, D). Besides, incubation of human TGF-β1 is sufficient to promote the expression of perisotin in CF (Additional file 2: Fig. S4E,G). Similar results were obtained in CF with Smad2/3 overexpression (Additional file 2: Fig. S4F, H and Additional file 2: Fig. S2E, F). Predictions using Jasper online databases identified three highly conserved sequences in the periostin promoter that was predicted to bind Smad2, and one highly conserved sequence in the periostin promoter that was predicted to bind Smad3 (Additional file 2: Fig. S5A–D). Interestingly, these prediction sequences were located in the activated region (− 1495 to − 1195) of periostin promoter. TGF-β1 stimulation, Smad2 overexpression, and Smad3 overexpression heightened the activity of periostin luciferase reporter gene (Additional file 2: Fig. S4I). Chromatin immunoprecipitation (ChIP) assay showed higher bindings of Smad2/3 to the periostin promoter in HG-challenged CF (Additional file 2: Fig. S4J, K), furthering confirming that the TGF-β/Smad pathway was required for HG to facilitate perisotin expression in CF.

### RNA sequencing revealed that perisotin was a diver of NAP1L2 in CF

**Fig. 5:**
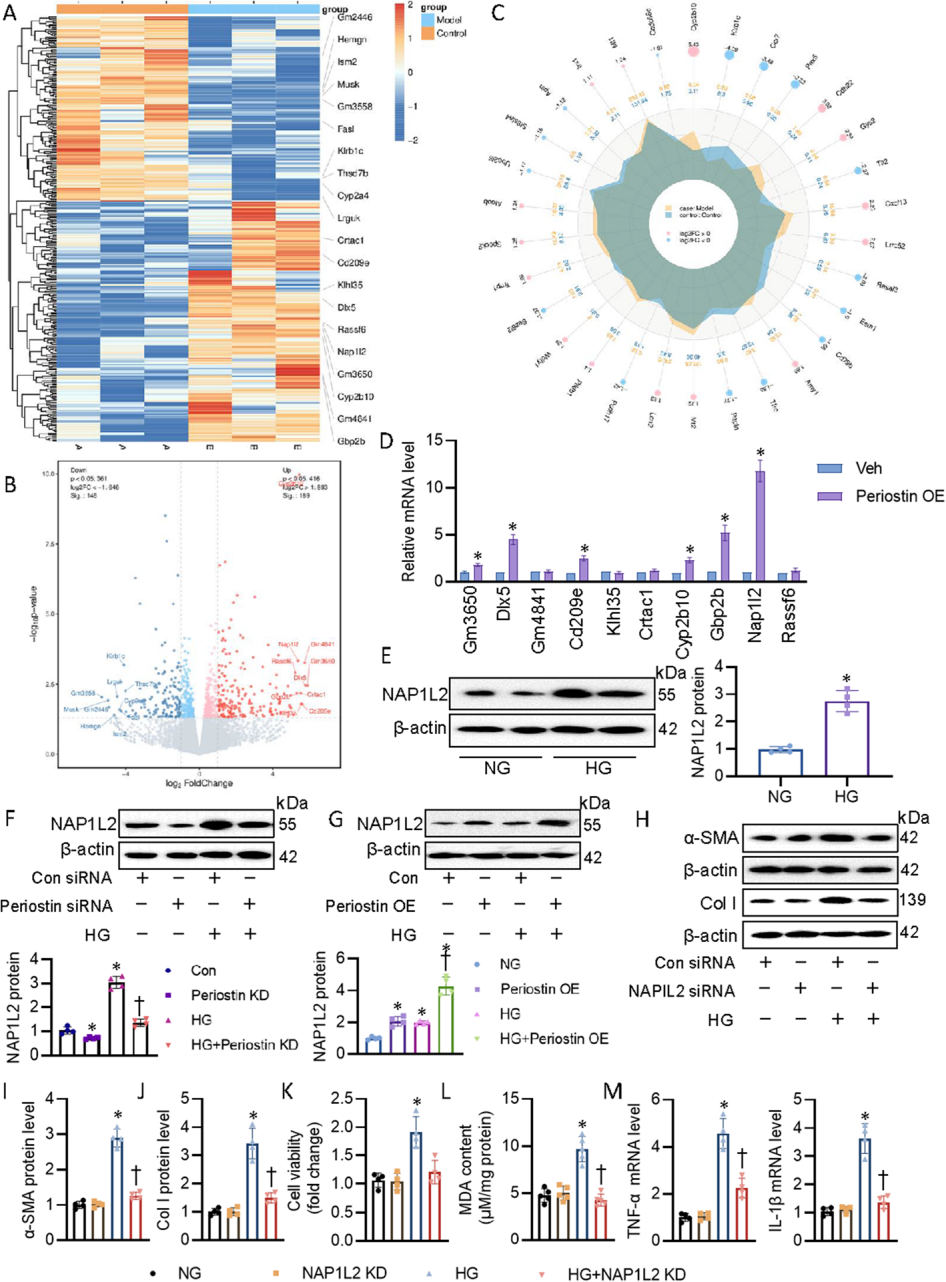
RNA sequencing revealed that perisotin was a diver of NAP1L2 in CF. **A** Heatmap showing the differential gene cluster analysis between control heart and periostin OE hearts. **B** Volcano map showing the overall distribution of differentially expressed genes between control heart and periostin OE hearts. **C** The differential gene expression level radar graph showed the 30 up/down genes with the lowest *Q* or *P* values. **D** The mRNA levels of top 10 upregulated genes in mouse hearts with periostin OE. **E** The protein expression of in CF exposed to NG or HG using western blot. **F** Effects of periostin siRNA on the protein expression of NAP1L2. **G** Effects of periostin OE on the protein expression of NAP1L2. **H–J** Representative blots and quantitation of α-SMA and collagen I. **K** Cell viability. **L** MDA contents. **M** Representative mRNA levels of TNF-α and IL-1β. *n* = 4–6. *P < 0.05 versus Vehicle (Veh), Con or NG, †*P* < 0.05 versus HG. The *P*-value was calculated by unpaired two-tailed Student’s *t*-test (**D**, **E**). Differences between groups were assessed with ANOVA followed by Bonferroni post-hoc test (**F**–**M**)

Next, we performed RNA sequencing to investigate the transcriptomic changes caused by periostin overexpression. The cluster analysis of differential gene expression level showed that 189 genes were upregulated and 146 genes were downregulated in CF after periostin overexpression (Fig. 5A). Volcano dots were used to display dysregulated genes between two groups, and all identified genes were clearly classified into two different cohorts (Fig. 5B). Radar map of differential gene expression level displayed the 30 upregulated or downregulated genes with the lowest Q or P value (Fig. 5C). We then conducted RT-PCR to examine the mRNA levels of the top 10 elevated genes, and found that periostin overexpression markedly upregulated the mRNA level of the nucleosome assembly protein 1-like 2 (NAP1L2) (Fig. 5D). In keeping with this, HG incubation boosted the protein expression of NAP1L2 (Fig. 5E). Western blotting analysis demonstrated a decreased protein level of NAP1L2 in periostin-knockdown CF under both NG and HG conditions (Fig. 5F). By contrast, upregulation of periostin not only raised the protein of NAP1L2, but also worsened HG-induced expression of NAP1L2, indicating that periostin is an upstream mediator for NAP1L2 (Fig. 5G). To further assess the functional role of NAP1L2 in CF activation, we used NAP1L2 siRNA to downregulate this protein. As expected, NAP1L2 downregulation rendered CF more invulnerable to HG-induced CF activation, as seen by lower fibrosis, oxidative stress, and inflammation (Fig. 5H–M), indicating that periostin is a positive regulator of NAP1L2, leading to CF activation in diabetes. As a class of small noncoding RNA molecules, miRNAs are established to induce the translation repression or mRNA degradation by binding to the 3’-untranslated regions (3’-UTRs) of a target mRNA sequence (9). It is interesting to know whether periostin positively regulated NAP1L2 expression in a miRNA-dependent manner. To this, end, the potential miRNAs that regulated NAP1L2 were explored using TargetScan, miRWalk, and miRDB databases. Venn diagrams are utilized to explore overlapping miRNAs, and results showed that these four databases share only one miRNA, miR-27b-3p (Additional file 2: Fig. S6A). The conserved sites for miRNA families broadly conserved among vertebrates were shown in Additional file 2: Fig. S6B. Further, we used dual-luciferase reporter gene assay to determine the interaction between miR-27b-3p and NAP1L2. Ectopic expression of miR-27b-3p significantly suppressed the luciferase activity of wild-type (WT) NAP1L2 reported gene, this was disappeared in mutant NAP1L2 reported gene (Additional file 2: Fig. S6C, D), suggesting that miR-30a-5p negatively regulated the expression of NAP1L2 in CFs. Importantly, inhibition of miR-30a-5p further potentiated, whereas upregulation of miR-30a-5p significantly reversed the actions of periostin overexpression on the protein of NAP1L2 (Additional file 2: Fig. S6E, F). These results indicated that periostin positively modulated the expression of NAP1L2 by dissociating the binding of miR-30a-5p to the 3’-UTRs of NAP1L2.

### NAP1L2 regulated SIRT3 in CFs

As is known, NAP1L2 is a histone chaperone that is involved in the dynamics of histone acetylation at its target loci during cellular differentiation [35]. The sirtuin (SIRT) deacetylases are important members that govern histone acetylation in both health and diseases [36]. We thus investigated whether NAP1L2 exerted its function through a similar epigenetic mechanism via regulating the SIRT family. The potential interactions between NAP1L2 and SIRT1-7 were detected by the co-IP, and results demonstrated that NAP1L2 could bind to SIRT1 and SIRT3, but not SIRT2, SIRT4, SIRT5, SIRT6 and SIRT7 (Additional file 2: Fig. S7A). Intriguingly, the decreased SIRT3 protein was markedly restored in HG-incubated CF upon NAP1L2 silencing (Additional file 2: Fig. S7B). On the contrary, deletion of NAP1L2 had no effect on HG-induced inhibition of SIRT1 in CF (Additional file 2: Fig. S7B). These observations indicated that perisotin upregulated NAP1L2 to recruit SIRT3 to affect CF functions in diabetes.

### Perisotin/NAP1L2 blunted BCAA catabolism in CF

**Fig. 6:**
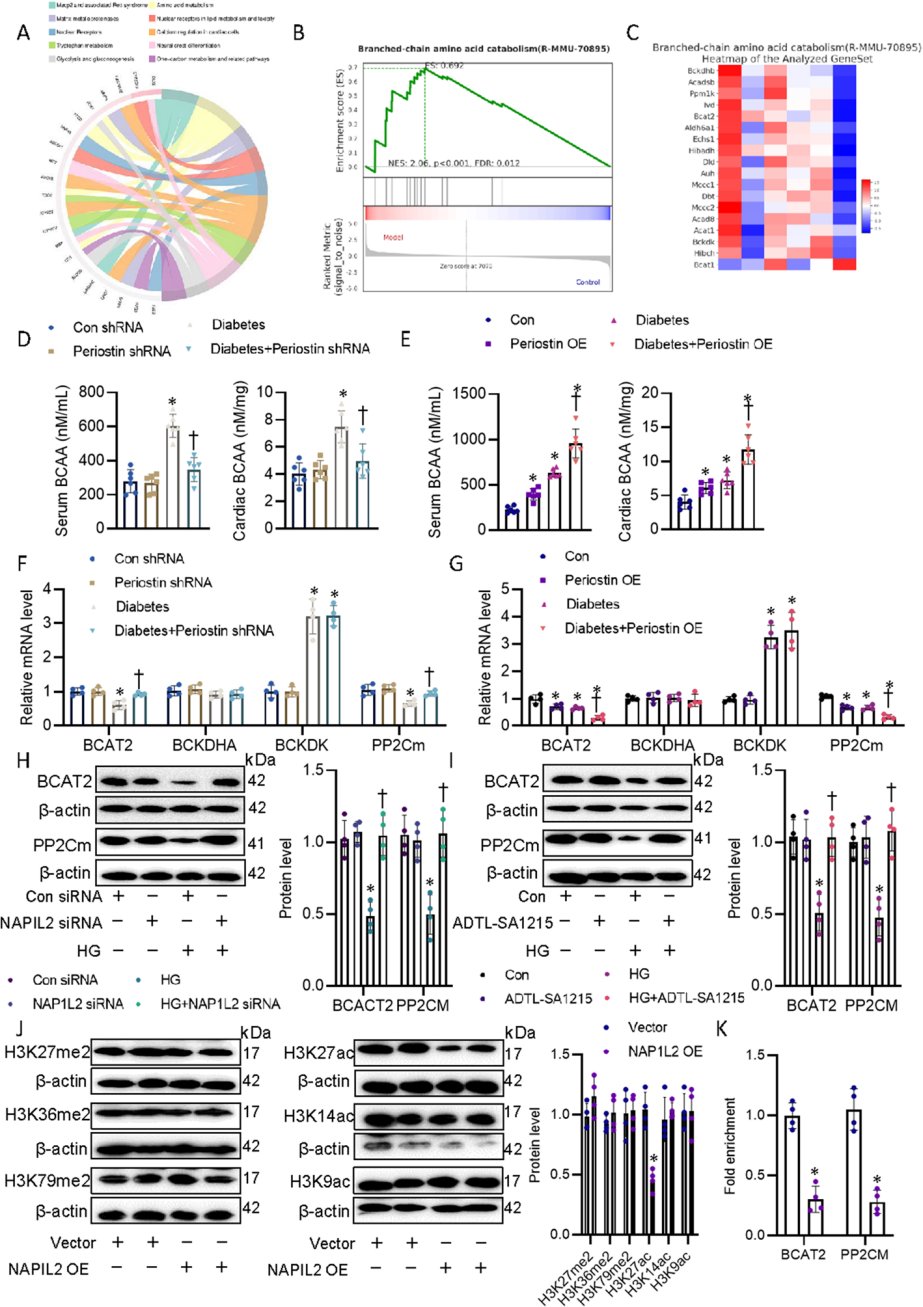
Perisotin/NAP1L2 blunted BCAA catabolism in CF. **A** Enrichment analysis chord diagram showing the BCAA catabolism was impaired in hearts after periostin OE. **B** Branched-chain amino acid catabolism (R-MMU-70895).gsea. **C** Branched-chain amino acid catabolism (R-MMU-70895) Heatmap of the Analyzed GeneSet. **D** Serum and cardiac BCAA levels in mice without periostin. **E** Serum and cardiac BCAA levels in mice with periostin OE. **F** Representative mRNA levels of BCAT2, BCKDHA, BCKDK, and PP2Cm in hearts from mice without periostin. **G** Representative mRNA levels of BCAT2, BCKDHA, BCKDK, and PP2Cm in hearts from mice with periostin OE. **H** Representative blots and quantitation of BCAT2 and PP2Cm in CF after silencing NAP1L2. **I** Representative blots and quantitation of BCAT2 and PP2Cm in CF after treatment with ADTL-SA1215 (5 μM), an agonist of SIRT3. **J** Representative blots and quantitation of H3K27me2, H3K36me2, H3K79me3, H3K27ac, H3K14ac, and H3K9ac in CF upon NAP1L2 overexpression. **K** H3K27acoccupancy at the promoters of BCAT2 and PP2Cm in CF transfected with NAP1L2 OE plasmid by ChIP. *n* = 3–4. *P < 0.05 versus Con, Con siRNA or Vector, †*P* < 0.05 versus HG or Diabetes. The *P*-value was calculated by unpaired two-tailed Student’s *t*-test (**K**). Differences between groups were assessed with ANOVA followed by Bonferroni post-hoc test (**D**–**I**)

Branched chain amino acids (BCAAs), consisting of leucine, isoleucine, and valine, are a group of essential amino acids that can be catabolized in the heart [37]. Alterations of BCAA metabolism have been found to lead to numerous prevalent diseases, including cardiometabolic diseases [38]. Aberrant accumulation of BCAAs in the heart is a driving force for cardiac pathological remodeling and heart failure [39]. Enrichment analysis chord diagram from RNA sequencing showed that periostin overexpression affected numerous signaling pathways, including amino acid metabolism (Fig. 6A). Gene Set Enrichment Analysis (GSEA) showed that overexpression of periostin affected BCAA catabolism (Fig. 6B). Bioinformatics analysis showed that periostin significantly influenced the levels of BCAAs catabolism-related genes (Fig. 6C). Targeted metabolomic analysis further identified that serum levels of leucine, valine, isoleucine were significantly higher in diabetic mice when compared with control mice (Additional file 2: Fig. S8). To further confirm whether periostin exerted the metabolism of BCAAs, we examined the levels of serum and cardiac BCAAs in mice. In accordance with transcriptomics, serum and cardiac levels of BCAAs were dramatically elevated in diabetic mice, an observation that was counteracted by the absence of periostin (Fig. 6D). Conversely, periostin overexpression mice had higher levels of serum and cardiac BCAAs, this phenomenon was further intensified in diabetic mice (Fig. 6E). The levels of BCAAs are mediated by tissue-specific inactivation of BCAA catabolizing enzymes, including branched-chain amino transferase 2 (BCAT2), branched-chain α–keto acid dehydrogenase (BCKDHA), branched-chain α–keto acid dehydrogenase kinase (BCKDK) and mitochondrial phosphatase 2C (PP2Cm) [40]. To examine the effects of periostin on BCAA catabolism, we examined the levels of the catabolic enzymes BCAT2, BCKDHA, BCKDK, and PP2Cm in the hearts. RT-PCR revealed lower mRNA levels of BCAT2 and PP2Cm, but higher mRNA level of BCKDK in diabetic hearts than that found in normal heart, such effects were reversed by deficiency of periostin and aggravated by overexpression of periostin (Fig. 6F, G), indicating that periostin might facilitate the impairment of BCAA catabolism in diabetic hearts through regulating BCAA catabolizing enzymes. Consistent with this, overexpression of BCAT2 or PP2Cm (Additional file 2: Fig. S9A, C, D, F) obviously prevent myofibroblast differentiation of CF after periostin overexpression, as indicated by measurement of Col I (Additional file 2: Fig. S9G). Conversely, knockdown of BCAT2 or PP2Cm (Additional file 2: Fig. S9B, C, E, F) markedly prevent myofibroblast differentiation of CF in the presence of periostin overexpression (Additional file 2: Fig. S9H). These findings revealed that impaired BCAA metabolism was required for the myofibroblast phenotype, leading to DCM-induced cardiac dysfunction.

Next, we determined whether the NAF1L2/SIRT3 axis had an impact on BCAA catabolism in diabetes. As expected, the protein expression of BCAT2 and PP2Cm were higher in NAF1L2-deficient or SIRT3-activated CF than those of control cells in response to HG stimuli (Fig. 6H, I and Additional file 2: Fig. S2G), indicating that the NAF1L2/SIRT3 pathway was involved in hyperglycemia-induced dysfunction of BCAA catabolism. As mentioned earlier, NAP1L2 and SIRT3 regulated cellular functions through histone acetylation modification, we hypothesized that NAP1L2 may recruit SIRT3 to induce deacetylation of promoters around the BCAT2 and PP2Cm. We screened the global status of the most common histone modifications upon NAP1L2 overexpression, including H3K27me2, H3K36me2, H3K79me3, H3K27ac, H3K14ac, and H3K9ac. We found only H3K27ac level was remarkably diminished in comparison with the non-target control (Fig. 6J). Moreover, the results of ChIP-qPCR demonstrated that the acetylation modification of H3K27 on the promoter regions of BCAT2 and PP2Cm was strikingly inhibited by overexpression of NAP1L2 (Fig. 6K), which would induce their downregulation and subsequent BCAA deposition in the heart. Intriguingly, overexpression of NAP1L2 prominently enhanced the acetylation modification of H3K27 on the promoter regions of known fibrotic genes, such as Col I and α-SMA (Additional file 2: Fig. S10). Totally, NAP1L2 might regulate BCAA catabolism enzymes and fibrotic proteins through H3K27 acetylation chromatin remodeling in the development of DCM.

### Chemical screening and identification of glucosyringic acid (GA), which directly targeted and inhibited perisotin expression in diabetic hearts

**Fig. 7:**
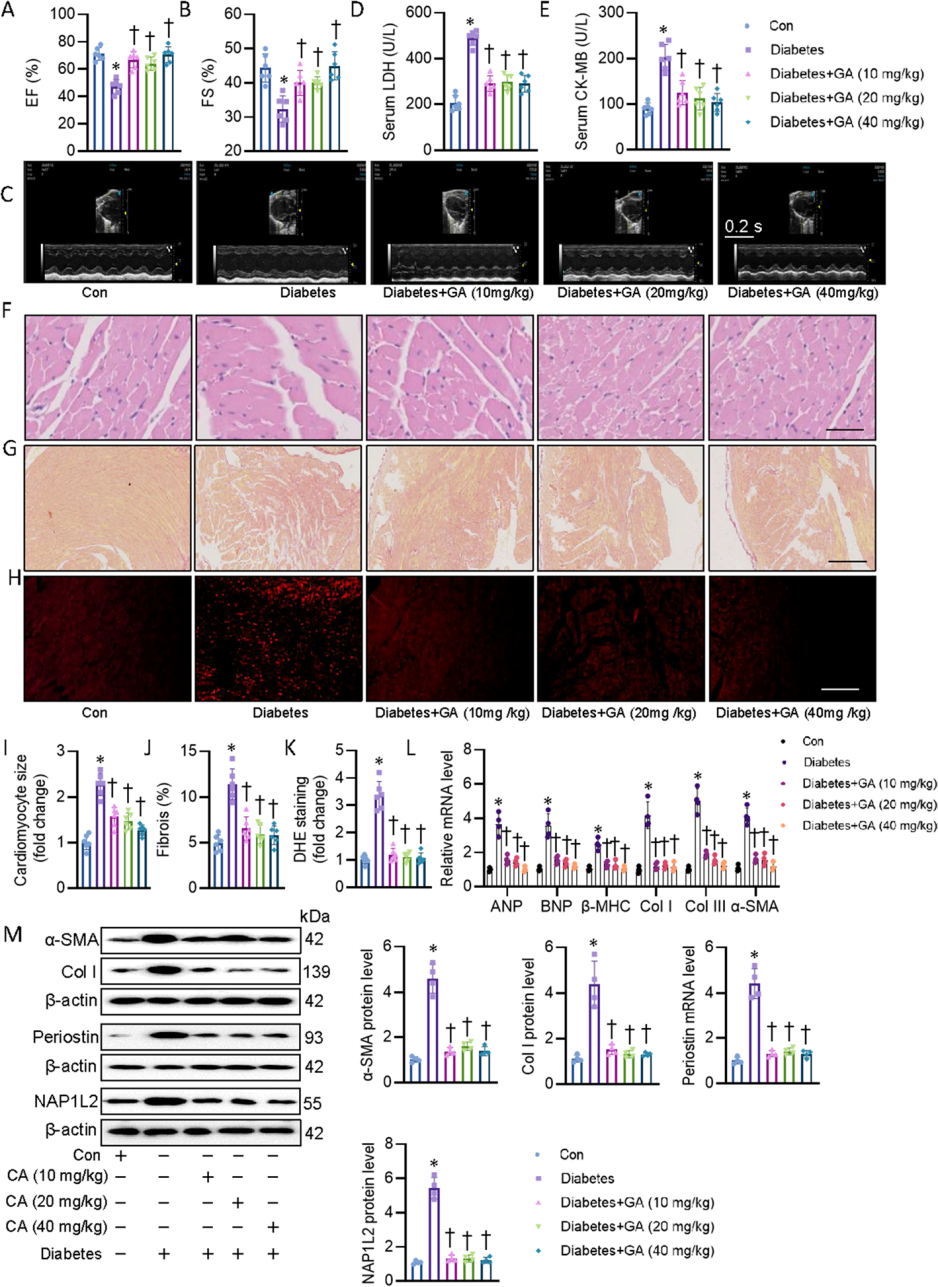
Chemical screening showing that GA improved DCM by directly targeted and inhibited perisotin expression in mice. **A**, **B** Left ventricle EF and FS were quantified. **C** Representative echocardiographic images showing the effects of perisotin knockdown on cardiac function in control and diabetic mice. **D** Serum LDH levels in mice. **E** Serum CK-MB levels in mice. **F**, **I** Representative photographs of the myocardium with H&E staining (Scale bar = 100 μm). **G**, **J** Representative photographs of the myocardium with Sirus red staining (Scale bar = 100 μm). **H**, **K** Representative images of the myocardium with DHE staining (Scale bar = 200 μm). **L** The mRNA levels of pro-hypertrophic genes (*ANP*, *BNP*, *β-MHC*) and pro-fibrogenic genes (*Col I*, *Col III*, *α-SMA*). **M** Representative blots and quantitation of α-SMA, collagen I, and NAP1L2. *n* = 4–6. *P < 0.05 versus Con, †*P* < 0.05 versus Diabetes. Differences between groups were assessed with ANOVA followed by Bonferroni post-hoc test (**D**–**I**)

Because of the critical role of periostin in DCM, we aimed to determine whether pharmacologic suppression of periostin would be a therapeutic approach for DCM. To screen the potential inhibitors of periostin, we established the CF expressing a luciferase reporter driven by a periostin-containing promoter. From a small molecule pool containing about 349 natural products, GA was found to significantly decrease the periostin-luciferase activity (Additional file 2: Fig. S11A–C). Molecular docking analysis revealed a direct interaction between GA and periostin with binding energy of − 6.6 kJ/mol (Additional file 2: Fig. S11D). In support, surface plasmon resonance (SPR) also confirmed the potential interaction of GA with periostin (Additional file 2: Fig. S11E). GA treatment had negligible cytotoxicity in CF, even at the higher concentrations (Additional file 2: Fig. S11F), and inhibited periostin protein and mRNA levels in HG-incubate CF (Additional file 2: Fig. S11G, H). Importantly, GA treatment increases CF resistance to HG-induced CF proliferation, oxidative stress, inflammation, and fibrosis (Additional file 2: Fig. S11I–L). Next, we assessed the pharmacological potential of GA in vivo in DCM mice. Results showed that cardiac function and injury from GA-treated mice were notably restored than those from control mice (Fig. 7A–E), while GA treatment had no effect on body weight and blood biochemical indicators (Additional file 1: Table S9). Likewise, histological analysis results indicated that heart from GA-treated mice exhibited lower cardiomyocyte size, fibrosis, oxidative stress (Fig. 7F–K), which was in line with decreased mRNA levels of hypertrophic and fibrogenic genes (Fig. 7L). Moreover, GA treatment repressed the protein expression of periostin, NAP1L2, α-SMA and Col (Fig. 7M). In addition, the protein expression of BCAT2, PP2Cm and SIRT3 was unsurprisingly restored by GA treatment in diabetic mice (Additional file 2: Fig. S12A). Accordingly, treatment of GA noticeably decreased circulating and cardiac BCAA contents in DCM mice (Additional file 2: Fig. S12B-C). Both in vivo and in vitro studies suggest that the mitigation of DCM in mice by GA may be dependent on periostin suppression.

## Discussion

Periostin is reported to participate in tissue injury and fibrotic remodeling [41]. Although the role of periostin in cardiac injury and myocardial fibrosis has been widely studied, the potential impact of periostin in DCM had been unexplored. Here, we found for the first time that deficiency of periostin overtly attenuated STZ/HFD-induced cardiac dysfunction and myocardial remodeling without affecting body weight, FBG, lipid and glucose metabolism in diabetic mice. Periostin-mediated dysfunction of myocardial structural and functional responses appeared to be dependent on NAP1L2, which induced inhibition of SIRT3 that deacetylates H3K27ac on the promoters of the BCAA catabolism-related enzymes BCAT2 and PP2CM, resulting in the accumulation of BCAAs in CF. These findings collectively support a deleterious role for periostin in diabetes-induced cardiac anomalies.

Cardiac fibrosis is characterized by interstitial and perivascular ECM accumulation, leading to myocardial remodeling [4, 42]. Activation of CF is a critical step involved in cardiac fibrosis since CF are recognized as the principal producer of ECM surrounding the CM [43]. CF play an important role in the development of fibrosis of DCM by remodeling of collagen and switching to a pro-fibrotic, highly contractile, myofibroblast phenotype [44, 45]. Inactivation of CF may be an attractive strategy for the treatment of cardiac fibrosis in DCM [46]. In this study, we found that the expression of periostin was upregulated in HG-exposed CF, not CM and EC, this was in line with the single cell sequencing results, indicating that CF are one of the main types of cardiac cells to generate periostin in DCM. Actually, we found that silencing perisotin attenuated, whereas upregulating periostin potentiated HG-induced proliferation, oxidative stress, and myofibroblast transformation of CF, suggesting that periostin aggravated cardiac fibrosis and dysfunction via myofibroblast transformation of CF. As a secretory protein, periostin in CF might affect CM functions in a paracrine-dependent manner since we found that loss of periostin ameliorated cardiac hypertrophy in diabetic mice. To this end, we examined whether CF-secreted periostin insulted biological behaviors of CM. Our results showed that CF-secreted periostin induced injury in CM as CM were prone to hypertrophy, apoptosis, and oxidative damage upon exposure of conditioned medium from CF with periostin overexpression. However, the molecular mechanism by which periodin secreted by CF caused myocardial cell hypertrophy is undefined in this study, which deserved further studies.

TGF-β/Smad and PI3K/Akt act as important signaling nodes in the pathogenesis of DCM [47, 48], which have been linked to the regulation of periostin [32, 33]. Thus, we examined whether the TGF-β/Smad and PI3K/Akt pathways would be required for periostin upregulation in diabetic hearts. Our results showed that blockade of the TGF-β/Smad axis prevented HG-induced upregulation of periostin in CF. However, inhibition of the PI3K/Akt axis did not affect the expression of periostin in HG-incubated CF. Coincidentally, luciferase reporter gene truncation results showed that a small region (− 1195 to − 895) of periostin promoter where Smad2/3 located was activated in CF in the presence of HG. In line with this, the binding of Smad2/3 to the periostin promoter was significantly enhanced in CF after HG challenge. Collectively, we prove that periostin is induced by TGF-β/Smad activation in diabetic hearts.

Next, we used transcriptomics on the molecular signaling pathways that may regulate the pathological cardiac dysfunction in diabetes induced by periostin. RNA sequencing results revealed that the transcriptional level of NAP1L2 was remarkably upregulated in CF with periostin overexpression. NAP1L2 plays a critical role in neural cell differentiation and mesenchymal stem cell senescence by regulating histone acetylation [35, 49]. Nevertheless, the potential roles and mechanisms of NAP1L2 in regulating CF activation have not yet been investigated. From functional analysis, we found that NAP1L2 exhibited higher expression in HG-stimulated CF, and knockdown of NAP1L2 ameliorated HG-induced proliferation, oxidative stress, inflammatory response and myofibroblast transformation of CF, indicating that NAP1L2 might be a stimulator for CF activation during the development of DCM.

Mounting evidence suggests that aberrant metabolism and accumulation of BCAAs play a crucial role in the initiation and development of cardiovascular and metabolic disorders [50–52], including DCM [53]. Bioinformatics analysis from RNA sequencing showed that periostin might disrupt the catabolic processes of BCAAs. Consistently, downregulation of periostin facilitated BCAA catabolism through upregulating BCAA catabolism-related enzymes BCAT2 and PP2Cm in diabetic hearts. Overexpression of periostin afforded the opposite effects. As a downstream mediator of periostin, it is unknown whether and how NAP1L2 is involved in the impairment of BCAA catabolism in DCM. Recently, NAP1L2 serves as a histone chaperone to recruited SIRT1 to deacetylate H3K14ac on promoters of osteogenic genes, thus leading to impaired osteogenic differentiation [49]. Our IP results showed that NAP1L2 interacted with SIRT1/3, but not other SIRT family members in CF. The protein expression of SIRT1/3 was significantly elevated in CF upon HG exposure, yet, only the protein expression of SIRT3 was restored by silencing NAP1L2, indicating that NAP1L2 might affect SIRT3 to affect CF functions in diabetes. It is of the utmost importance to mention that NAP1L2 knockdown and SIRT3 activation also elevated the protein expression of BCAT2 and PP2Cm in HG-exposed CF, indicating that periostin might decrease BCAA catabolism in DCM by regulating the NAP1L2/SIRT3 pathway. Functional studies showed that NAP1L2 was involved in restraining H3K27 acetylation (H3K27ac) on promoters of BCAA catabolic enzymes BCAT2 and PP2Cm. Overexpression of NAP1L2 significantly inhibited the global H3K27ac and NAP1L2 actually recruited SIRT3 which reduced the level of H3K27ac around the promoters of BCAT2 and PP2Cm, thereby leading to catabolic defect of BCAAs during the pathogenesis of DCM. Of note, it is questionable that a change in CF BCAA metabolism would impact serum or whole heart metabolism as it only comprises < 1–5% of the total volume of the heart. It seems more plausible that the alterations in cardiac BCAA metabolism/catabolism to the whole heart are simply a consequence of worsened cardiac disease state. It is believed that impaired BCAA catabolism in cardiac fibroblast (CF) might may also affect the BCAA metabolism of other cardiac cells, which needs more solid evidence in future studies.

Considering that periostin may be an important player in the pathological processes of DCM, the potential inhibitors of perisotin were thus important tools in developing effective treatment for DCM. By screening our phytochemical compound library based on the luciferase reporter gene activity of periostin, we identified that the phytochemical GA directly bound to periostin, inhibited the protein expression of periostin. Cellular and animal studies consistently demonstrated that GA treatment obviously improved DCM pathologies by suppressing the expression of periostin and restoring normal catabolism of BCAAs. Altogether, these findings indicated that GA may be a potential candidate compound for DCM therapy.

## Conclusions

Taken together, functional characterization of the periostin/NAP1L2/SIRT3 axis dysregulation and subsequent BCAA catabolism impairment would provide mechanistic insights into the role of periostin in DCM progression. The translational merit of our study is to reveal that the phytochemical GA had an inhibitory effect on periostin expression, serving as a promising drug candidate for the management of DCM.

### Limitations

Although we concluded that manipulating periostin expression may be a promising strategy for DCM treatment, it is crucial to note that translating findings from basic research to clinical applications can be complex and may require extensive further research, including clinical trials. Moreover, the dose of STZ used (120 mg/kg) in this study is too high to be considered type 2 diabetes (T2D) [54] although there are literatures that use high dose STZ and high-fat diet to simulate T2D. It is important to consider the relevance of this animal model to human DCM, as findings in mice may not always directly translate to humans. Future studies are warranted to determine the specific role of periostin in cardiac injury of T2D mice, such as genetically T2D (ob/ob, db/db) mice. In addition, the precise molecular mechanisms of periostin in DCM may require further validation and clarification. More importantly, periostin fibroblast-specific knockout and transgenic mice were warranted to determine the specific role of periostin in the pathogenesis of DCM.

### Supplementary Information

**Additional file 1**: **Table S1**. Information for primary and secondary antibodies. **Table S2**. Primers for Real-time quantitative PCR analysis in mice. **Table S3**. Primers for Real-time quantitative PCR analysis in cells. **Table S4**. Compound information used for screening experiments. **Table S5**. Comparison of clinical data of enrolled participates and patients. **Table S6**. Primers for ChIP analysis in the present study. **Table S7**. Biochemical characteristics and echocardiographic data of control and diabetic mice in the presence or absence of periostin. **Table S8**. Biochemical characteristics and echocardiographic data of control and diabetic mice after overexpression of periostin. **Table S9**. Biochemical characteristics and echocardiographic data of control and diabetic mice after treatment of GA.

**Additional file 2**: **Fig. S1**. Effects of HG, TGF-β1 and Ang II on the mRNA level of periostin in CF. **Fig. S2**. Expression of target proteins after knockdown or overexpression. **Fig. S3**. Cardiac fibroblasts-secreted periostin induced hypertrophy and apoptosis in primary neonatal cardiomyocytes. **Fig. S4**. Periostin is induced by HG in a TGF-β/Smad dependent manner. **Fig. S5**. Binding of Smad2/3 to the promoters of periostin. **Fig. S6**. Periostin upregulated NAP1L2 via suppressing miR-27b-3p. **Fig. S7**. NAP1L2 regulated SIRT3 in CF. **Fig. S8**. Heatmap of mean frailty score in quartiles of serum BCAAs in control and diabetic mice. **Fig. S9**. Effects of BCAT2 and PP2Cm on the myofibroblast differentiation of CF. **Fig. S10**. H3K27acoccupancy at the promoters of α-SMA and Col I in CF transfected with NAP1L2 OE plasmid by ChIP. **Fig. S11**. Effects of different compounds on periostin luciferase reporter activity and expression. **Fig. S12**. Effects of GA on the BCAA catabolism in mouse hearts.

## Data Availability

The data and materials in the current study are available from the corresponding author on reasonable request.

## Abbreviations

β-MHC: Beta-myosin heavy chain
ANP: Atrial natriuretic peptide
BCAAs: Branched-chain amino acids
BCAT2: Branched-chain amino transferase 2
BCKDHA: Branched-chain α-keto acid dehydrogenase
BCKDK: Branched-chain α-keto acid dehydrogenase kinase
BMP: Bone morphogenetic protein
BNP: Brain natriuretic peptide
CF: Cardiac fibroblasts
ChIP: Chromatin immunoprecipitation
CK-MB: Creatine kinase-MB
DCM: Diabetic cardiomyopathy
ECM: Extracellular matrix
ELISA: Enzyme-linked immunosorbent assay
FBG: Fasting blood glucose
GA: Glucosyringic acid
GSEA: Gene Set Enrichment Analysis
HEK: Human embryonic kidney
HFD: High-fat diet
HG: High glucose
HOMA-IR: Homeostatic model assessment for insulin resistance
IL-1β: Interleukin 1β
LDH: Lactate dehydrogenase
NAP1L2: Nucleosome assembly protein 1-like 2
NG: Normal glucose
PP2Cm: Phosphatase 2C
ROC: Receiver-operating characteristic
RT-PCR: Reverse transcription polymerase chain reaction
SD: Standard deviation
SIRT3: Sirtuin 3
STZ: Streptozotocin
TGF-β: Transforming growth factor-β
TNF-α: Tumor necrosis factor α
