# Supplementary material for: Disrupted cardiac fibroblast BCAA catabolism contributes to diabetic cardiomyopathy via a periostin/NAP1L2/SIRT3 axis
Source: Cell Mol Biol Lett. 2023 Nov 22;28:93. doi: 10.1186/s11658-023-00510-4 (PMC10666354; doi:10.1186/s11658-023-00510-4)
Supplement: Supplementary file 1 — Additional file 1: Table S1. Information for primary and secondary antibodies. Table S2. Primers for Real-time quantitative PCR analysis in mice. Table S3. Primers for Real-time quantitative PCR analysis in cells. Table S4. Compound information used for screening experiments. Table S5. Comparison of clinical data of enrolled participates and patients. Table S6. Primers for ChIP analysis in the present study. Table S7. Biochemical characteristics and echocardiographic data of control and diabetic mice in the presence or absence of periostin. Table S8. Biochemical characteristics and echocardiographic data of control and diabetic mice after overexpression of periostin. Table S9. Biochemical characteristics and echocardiographic data of control and diabetic mice after treatment of GA. [file 11658_2023_510_MOESM1_ESM.docx]

**Additional file 1: Table S1. Information for primary and secondary antibodies.**

| Target | Company | Cat. No. | Dilution ratio |
| --- | --- | --- | --- |
| Periostin  Periostin  α-SMA  β-actin  Col I  NAP1L2 | Proteintech  Proteintech  Proteintech  Abcam  Abcam  Absin | 19899-1-AP  19899-1-AP   14395-1-AP  ab7817  ab270993  abs103449 | 1: 1,000 for WB  1:200 for IF  1: 1,000 for WB  1: 1,000 for WB  1: 1,000 for WB  1: 1,000 for WB |
| HA | Proteintech | 51064-2-AP | 1: 1,000 for WB |
| Flag | Proteintech | 66008-4-Ig | 1: 1,000 for WB |
| SIRT1  SIRT3  BCAT2  PP2Cm  H3K27me2  H3K36me2  H3K79me2  H3K27ac  H3K14ac  H3K9ac | Abcam  Abcam  Proteintech  Proteintech  Abcam  Abcam  Abcam  Abcam  Abcam  Abcam | ab110304  ab189860  16417-1-AP  14573-1-AP  ab24684  ab176921  ab177185  ab4729  ab203952  ab32129 | 1: 1,000 for WB  1: 1,000 for WB  1: 1,000 for WB  1: 1,000 for WB  1: 1,000 for WB  1: 1,000 for WB  1: 1,000 for WB  1: 1,000 for WB  1: 1,000 for WB  1: 1,000 for WB |
| Smad2  Smad3  α-actinin | Abcam  Abcam  Abcam | ab40855  ab208182  ab68194 | 1: 1,000 for WB  1: 1,000 for WB  1: 200 for IF |
| Anti-mouse HRP secondary antibody | Immunoway | RS0001 | 1:5,000 |
| Anti-rabbit HRP secondary antibody  [Goat Anti-Rabbit IgG H&L (Alexa Fluor® 488)](https://www.abcam.cn/products/secondary-antibodies/goat-rabbit-igg-hl-alexa-fluor-488-ab150077.html)  [Goat Anti-Rabbit IgG H&L (Alexa Fluor® 594)](https://www.abcam.cn/products/secondary-antibodies/goat-rabbit-igg-hl-alexa-fluor-488-ab150077.html) | Immunoway  Abcam  Abcam | RS0002  [ab150077](https://www.abcam.cn/products/secondary-antibodies/goat-rabbit-igg-hl-alexa-fluor-488-ab150077.html)  ab150080 | 1:5,000  1:1000  1:1000 |

**Additional file 1: Table S2. Primers for Real-time quantitative PCR analysis in mice**.

| **Gene name** | **Sequences** |
| --- | --- |
| Mouse Periostin | 5′-TGGTATCAAGGTGCTATCTGCG -3′  5′-AATGCCCAGCGTGCCATAA-3′ |
| Mouse ANP | 5′-ACCTGCTAGACCACCTGGAG-3′  5′-CCTTGGCTGTTATCTTCGGTACCGG-3′ |
| Mouse BNP | 5′-GAGGTCACTCCTATCCTCTGG-3′  5′-GCCATTTCCTCCGACTTTTCTC-3′ |
| Mouse β-MHC | 5'-CCGAGTCCCAGGTCAACAA-3'  5'-CTTCACGGGCACCCTTGGA-3' |
| Mouse α-SMA | 5′-GTGACTCACAACGTGCCT-3′  5′-CAGCCAAGTCCAGACGCAT-3′ |
| Mouse Collagen I | 5′-CCCAAGGAAAAGAAGCACGTC-3′  5′-AGGTCAGCTGGATAGCGACATC-3′ |
| Mouse Collagen III | 5′-TGGTAGAAAGGACACAGAGGC-3′  5′-TCCAACTTCACCCTTAGCACC-3′ |
| Mouse IL-1β | 5′-GGGGCGTCCTTCATATGTGT-3′  5′-ATACAACGGCTCCTCCGTTC-3′ |
| Mouse IL-6 | 5′-GAGGATACCACTCCCAACAGACC-3′  5′-AAGTGCATCATCGTTGTTCATACA-3′ |
| Mouse TNF-α | 5′-TGATCCGCGACGTGGAA-3′  5′-ACCGCCTGGAGTTCTGGAA-3′ |
| Mouse NOX2 | 5′- ACTCCTTGGGTCAGCACTGG-3′  5′- GTTCCTGTCCAGTTGTCTTGG-3′ |
| Mouse NOX4 | 5′-TGTTGGGCCTAGGATTGTGTT-3′  5′-AGGGACCTTCTGTGATCCTCG-3′ |
| Mouse BCAT2 | 5′-GTCATCTTGCCTGGAGTAGTTCG-3′  5′-TTGCTTGCCTTCATACAGGATTT-3′ |
| Mouse BCKDK | 5′-CGTAGCCTTCCTTTCATCATTG-3′  5′-CCTCATCTGCCTGGTCCTTG-3′ |
| Mouse PP2Cm | 5′-GCCAGGTGTTCTCGGTTTGA-3′  5′-TGGTTTGCCGTACTTGATGC-3′ |
| Mouse BCKDHA | 5′-AGGGCCGGATCTCCTTCTACAT-3′  5′-CCTTGCCTGGGTCATTCACG-3′ |
| Mouse 12-lox | 5′-TCCAGTGGCTACGAGGATCT-3′  5′-GGCGGGGGCAATTCAAATAC-3′ |
| Mouse Gm3650 | 5′-ATAAAAGGGACTGGCCAGGC-3′  5′-GCCTGGCCAGTCCCTTTTAT-3′ |
| Mouse Dlx5 | 5′-CACCACCCGTCTCAGGAATC-3′  5′-GCTTTGCCATAAGAAGCAGAGG-3′ |
| Mouse Gm4841 | 5′-AGTGCCTGTTGAACTGTTGATAA-3′  5′-CCAGGAGCCCACCATTAGC-3′ |
| Mouse Cd209e | 5′-CCACATTCCCCTGGTGTTG-3′ 5′-CAGAGGCGACAGAGTCTATCA-3′ |
| Mouse Klhl35 | 5′-TATACACGCTCTGAATTTGCCTC-3′  5′-GATGGGAGCTAAACATCCAGAC-3′ |
| Mouse Crtac1 | 5′-GTGACAGATGTGGACCATGATG-3′  5′-CTGGGCTCGATTGTACTTCAG-3′ |
| Mouse Cyp2b10 | 5′-TTAGTGGAGGAACTGCGGAAA-3′  5′-CGCAAGAACTGACGGTCTG-3′ |
| Mouse Gbp2b | 5′-CCCAGTGTGCCTCATCAAGAA-3′  5′-GCTACGACCACCACAGGATTTT-3′ |
| Mouse Nap1l2 | 5′-GACGACAGATCATCAATGCAGT-3′  5′-TTGCCGTTAGTCTCTTCCTCC-3′ |
| Mouse Rassf6 | 5′-GACCTTTACCGTATCAGCGAG-3′  5′-GCGTATGGTGTCAGAGTGCT-3′ |
| Mouse β-actin | 5′-CCGTGAAAAGATGACCCAGA-3′  5′-TACGACCAGAGGCATACAG-3′ |

**Additional file 1: Table S3. Primers for Real-time quantitative PCR analysis in cells**.

| **Gene name** | **Sequences** |
| --- | --- |
| Rat Periostin | 5′-CCCTCCCACTGCTAGGCTAT-3′  5′-GTGGTCGACTTACCTGAAGCG-3′ |
| Rat ANP | 5′-GGCCTTTTGGCTCCCAGGCC-3′  5′-CTAAGTGCCGCCCCCGCTTC-3′ |
| Rat BNP | 5′-TTGGGCAGAAGATAGACCGGAT-3′  5′-GGTCTTCCTAAAACAACCTCA-3′ |
| Rat β-MHC | 5'-GCAGACAGAGAATGGGGAGCTGTCC-3'  5'-TCGCAATCATGCCGGGCTGAC-3' |
| Rat α-SMA | 5′- CTTGGCCCTGTTGTGTTCTCT-3′  5′-TAAAAGAGCCTGTGACGCTCC-3′ |
| Rat Collagen I | 5′-GACATCCCTGAAGTCAGCTGC-3′  5′-TCCCTTGGGTCCCTCGAC-3′ |
| Rat IL-1β | 5′-TTGCCATAGCTGCTTCAGACA-3′  5′-TGCCCTGAACTAGAGCTGAGA-3′ |
| Rat IL-6 | 5′-GAGTTGTGCAATGGCAATTC-3′  5′-ACTCCAGAAGACCAGAGCAG-3′ |
| Rat TNF-α | 5′-TACTCCCAGGTTCTCTTCAAGG-3′  5′-GGAGGCTGACTTTCTCCTGGTA-3′ |
| Rat NOX2 | 5′-ACCAGCTGCACAAACAGAAAA-3′  5′-GTCTGAACTTGGGGAAGTCCT-3′ |
| Rat 12-LOX | 5′-CAGCTTACCGAGTCCCGACA-3′  5′-TACCTGTGGTTGGTTGGACA-3′ |
| Rat β-actin | 5′-AAGTCCCTCACCCTCCCAAAAG-3′  5′-AAGCAATGCTGTCACCTTCCC-3′ |

**Additional file 1: Table S4**. **Compound information used for screening experiments.**

| **Category** | **No.** | **Compounds** |
| --- | --- | --- |
| Benzimidazoles | 1 | 5,6-Dimethylbenzimidazole |
| Carboxylic acids | 2 | 1-Naphthaleneacetic acid |
|  | 3 | 10-Hydroxydecanoic Acid |
|  | 4 | D-(+)-Galacturonic acid |
|  | 5 | Mandelic acid |
|  | 6 | Indole-3-acetic acid |
|  | 7 | Oxalic acid |
|  | 8 | 4-Methoxysalicylic acid |
| Curcuminoid | 9 | Bisdemethoxycurcumin |
| Flavonoids | 10 | Oroxin A |
|  | 11 | Procyanidin B1 |
|  | 12 | Hispidulin |
|  | 13 | 4',6,7-Trimethoxyisoflavone |
|  | 14 | Rhamnocitrin |
|  | 15 | Hamaudol |
|  | 16 | Gossypin |
|  | 17 | Dihydrodaidzein |
|  | 18 | Aromadendrin |
|  | 19 | 6,7,4'-Trihydroxyisoflavone |
|  | 20 | 6-Methoxyluteolin |
|  | 21 | 8-Prenylnaringenin |
|  | 22 | Sakuranetin |
|  | 23 | Skullcapflavone II |
|  | 24 | 4',5,7-Trimethoxyflavone |
|  | 25 | (±)-Catechin hydrate |
|  | 26 | 4'-METHOXYFLAVONE |
|  | 27 | 3,4-Dihydroxyflavone |
|  | 28 | 5,7,3',4'-Tetramethoxyflavone |
|  | 29 | Norwogonin |
|  | 30 | Kaempferol 3-gentiobioside |
|  | 31 | Visnagin |
|  | 32 | 5-Hydroxyflavone |
|  | 33 | Aloeresin D |
|  | 34 | EGCG Octaacetate |
|  | 35 | Tilianin |
|  | 36 | 6-Demethoxytangeretin |
|  | 37 | Quercetagetin |
|  | 38 | 4-METHOXYCHALCONE |
|  | 39 | 7-Hydroxyflavanone |
|  | 40 | 3-Methoxyflavone |
|  | 41 | 4-Hydroxyflavanone |
|  | 42 | 2-Hydroxyflavanone |
|  | 43 | 3,6-Dihydroxyflavone |
|  | 44 | 2-Hydroxychalcone |
|  | 45 | 4'-Hydroxychalcone |
|  | 46 | 7-Methoxyflavone |
|  | 47 | Reynoutrin |
|  | 48 | Theaflavin |
|  | 49 | 7-Hydroxyflavone |
|  | 50 | Procyanidin B2 |
|  | 51 | Apigenin-7-glucuronide |
|  | 52 | Sophoraflavanone G |
|  | 53 | Kurarinone |
|  | 54 | Kaempferol 3-glucorhamnoside |
|  | 55 | MOSLOFLAVONE |
|  | 56 | 5,7-DIMETHOXYFLAVONE |
|  | 57 | 5-hydroxy-7,8-dimethoxyflavone |
|  | 58 | Vitexia-glucoside |
|  | 59 | 5-DEMETHYLNOBILETIN |
|  | 60 | 2''-O-β-L-Galorientin |
|  | 61 | GALANGIN-3-METHYLETHER |
|  | 62 | 4',7-DIMETHOXY-5-HYDROXYFLAVONE |
|  | 63 | Maltol |
|  | 64 | Cyanidin Chloride |
|  | 65 | 3’- Methoxy Puerarin |
|  | 66 | Trifolirhizin |
|  | 67 | Quercetin-3-O-β-D-glucose-7-O-β-D-gentiobiosiden |
|  | 68 | HOMOPLANTAGININ |
|  | 69 | Isoliquiritin apioside |
|  | 70 | 7,2'-dihydroxy-3',4'-dimethoxyisoflavane-7-O-glucoside |
|  | 71 | Karanjin |
|  | 72 | 4-Hydroxycoumarin |
|  | 73 | Quercetagitrin |
|  | 74 | Procyanidin C1 |
|  | 75 | Hesperetin 7-O-glucoside |
|  | 76 | Iristectorigenin A |
|  | 77 | Isorhamnetin-3-O-glucoside |
|  | 78 | Quercimeritrin |
|  | 79 | Luteolin-3-O-beta-D-glucuronide |
|  | 80 | Neoeriocitrin |
|  | 81 | Taxifolin 7-O-rhamnoside |
|  | 82 | Vicenin 3 |
|  | 83 | Vaccarin |
| Glycerolipids | 84 | Glycerol Tri-n-octanoate |
|  | 85 | Glycerol Trieicosanoate |
|  | 86 | Glycerol Tridecanoate |
|  | 87 | Glycerol trilinoleate |
|  | 88 | 1-Oleoyl-rac-glycerol |
| Phenols | 89 | Cannabidiol |
|  | 90 | Hexahydrocurcumin |
|  | 91 | Dimethylcurcumin |
|  | 92 | Chebulinic acid |
|  | 93 | Chebulagic acid |
|  | 94 | 2',3'-Dihydroxy-4'-methoxyacetophenone |
|  | 95 | Homovanillyl alcohol |
|  | 96 | Homogentisic acid |
|  | 97 | Eugenin |
|  | 98 | Atranorin |
|  | 99 | Isopsoralenoside |
|  | 100 | Psoralenoside |
|  | 101 | Pyromeconic acid |
|  | 102 | Gigantol |
|  | 103 | Chicoric Acid |
|  | 104 | Nordihydroguaiaretic acid |
|  | 105 | Deoxyrhapontin |
|  | 106 | 2-5-dihydroxyacetophenone |
|  | 107 | Glucosyringic acid |
|  | 108 | Phenylacetaldehyde |
|  | 109 | 2'-Hydroxyacetophenone |
|  | 110 | BENZYLACETONE |
|  | 111 | trans-Benzylideneacetone |
|  | 112 | Atraric acid |
|  | 113 | 4-Methoxybenzoic acid |
|  | 114 | Forsythoside I |
|  | 115 | Raspberry ketone glucoside |
|  | 116 | 2-HYDROXY-3,4-DIMETHOXYBENZOIC ACID |
|  | 117 | androsin |
|  | 118 | Hydroxytyrosol Acetate |
|  | 119 | Cannabidivarin |
|  | 120 | Gallic aldehyde |
|  | 121 | Anisic aldehyde |
|  | 122 | 2-methoxycinnamaldehyde |
|  | 123 | 3,5-Dimethoxyphenol |
|  | 124 | 6-paradol |
|  | 125 | Gnetol |
|  | 126 | Ginkgolic acid C15:1 |
|  | 127 | Ginkgolic acid C13:0 |
|  | 128 | Ginkgolic acid C17:1 |
|  | 129 | Geraniin |
|  | 130 | Curculigoside |
|  | 131 | 6-Shogaol |
|  | 132 | 8-Gingerol |
|  | 133 | Paeonolide |
|  | 134 | Mulberroside A |
|  | 135 | Vanillin |
|  | 136 | Gossypol |
|  | 137 | Acetovanillone |
|  | 138 | D-DELTA-TOCOPHEROL |
|  | 139 | Orcinol gentiobioside |
|  | 140 | Apiopaeonoside |
|  | 141 | Desmethoxy yangonin |
|  | 142 | Rhaponiticin |
|  | 143 | 4-Ethylphenol |
|  | 144 | Isovanillic acid |
|  | 145 | 4'-Methoxyresveratrol |
|  | 146 | Methylnissolin-3-O-glucoside |
|  | 147 | Acetyl-trans-resveratrol |
|  | 148 | 3,4-Dimethoxybenzaldehyde |
|  | 149 | Zearalenone |
|  | 150 | 4-Hydroxymandelic acid |
|  | 151 | DL -3,4-Dihydroxymandelic acid |
|  | 152 | Agrimol B |
|  | 153 | Dryocrassin ABBA |
|  | 154 | Alnustone |
|  | 155 | DL-Normetanephrine hydrochloride |
|  | 156 | alpha-Arbutin |
|  | 157 | 3,4-Dihydroxyphenylacetic acid |
|  | 158 | 3-Hydroxyphenylacetic acid |
|  | 159 | 4-Methylcatechol |
|  | 160 | 3-Methoxytyramine hydrochloride |
|  | 161 | L-KAWAIN |
|  | 162 | Kakuol |
|  | 163 | Dendrophenol |
|  | 164 | Oxyresveratrol |
|  | 165 | yangonin |
|  | 166 | Thymol |
|  | 167 | Homovanillic acid |
|  | 168 | Punicalagin |
|  | 169 | 10-Gingerol |
|  | 170 | Erianin |
|  | 171 | 7,2'-Dihydroxy-3',4'-dimethoxyisoflavan |
|  | 172 | Pinosylvin |
|  | 173 | Dihydroresveratrol |
|  | 174 | Isorhapontigenin |
|  | 175 | Corilagin |
|  | 176 | 1,2,3,4,6-O-Pentagalloylglucose |
|  | 177 | Rhapontigenin |
|  | 178 | 2'-Hydroxy-5'-methoxyacetophenone |
|  | 179 | 2,6-Dimethoxybenzoic acid |
|  | 180 | Pinostilbene |
|  | 181 | 4-Hydroxybenzyl alcohol |
|  | 182 | Veratric acid |
|  | 183 | Olivetol |
|  | 184 | Tetrahydro Curcumin |
|  | 185 | Methyl gallate |
|  | 186 | Ethyl gallate |
|  | 187 | Methylparaben |
|  | 188 | Methyl syringate |
|  | 189 | β-thujaplicin |
|  | 190 | Sesamol |
|  | 191 | Helicid |
|  | 192 | 3,4-Dihydroxyphenylethanol |
|  | 193 | 6-Gingerol |
|  | 194 | Bakuchiol |
|  | 195 | Protocatechualdehyde |
|  | 196 | Honokiol |
|  | 197 | p-Hydroxybenzaldehyde |
|  | 198 | 5-Hydroxy-1,7-diphenyl-6-hepten-3-one |
|  | 199 | Isoeugenol |
|  | 200 | Chrysophanic Acid |
|  | 201 | Cardamonin |
|  | 202 | 4-Hydroxybenzoic acid |
|  | 203 | (-)-Epigallocatechin Gallate |
|  | 204 | Epigallocatechin |
|  | 205 | Xanthoxyline |
|  | 206 | Sodium Danshensu |
|  | 207 | 3,4,5-Trimethoxyphenol |
|  | 208 | Ethyl Vanillate |
|  | 209 | Paeonol |
|  | 210 | Pterostilbene |
|  | 211 | Phloretic acid |
|  | 212 | Tyrosol |
|  | 213 | Gentisic acid |
|  | 214 | Phloracetophenone |
|  | 215 | Orsellinic acid |
|  | 216 | Morin |
|  | 217 | Ethyl ferulate |
|  | 218 | Caffeic Acid |
|  | 219 | 7-Methoxy-4-methylcoumarin |
|  | 220 | Orsellinic acid ethyl ester |
|  | 221 | (+)-Catechin Hydrate |
|  | 222 | Orcinol glucoside |
|  | 223 | Rosmarinic acid |
|  | 224 | Gossypol acetic acid |
|  | 225 | Salvianolic acid B |
|  | 226 | Methylarbutin |
|  | 227 | Hematoxylin |
|  | 228 | Vitamin E |
|  | 229 | Resveratrol |
|  | 230 | Methyl protocatechuate |
|  | 231 | Terphenyllin |
|  | 232 | Guaiacol |
|  | 233 | Eugenol |
|  | 234 | Vitamin E Acetate |
|  | 235 | 3-Hydroxy-4-methoxyacetophenone |
|  | 236 | Gallic acid |
|  | 237 | Ethylparaben |
|  | 238 | Benzoic acid |
|  | 239 | Cianidanol |
|  | 240 | Tannic acid |
|  | 241 | 3,4-Dimethoxybenzyl alcohol |
|  | 242 | Gallic acid trimethyl ether |
|  | 243 | Methyl EudesMate |
|  | 244 | Protocatechuic acid |
|  | 245 | Ellagic acid |
|  | 246 | Phenylephrine hydrochloride |
|  | 247 | Methyl salicylate |
|  | 248 | Salicylamide |
|  | 249 | Methylsyringol |
|  | 250 | 4-Hydroxyphenylacetonitrile |
|  | 251 | 2'-Hydroxy-4'-methylacetophenone |
|  | 252 | Ethyl 4-hydroxyphenylacetate |
|  | 253 | 4-(4-Methoxyphenyl)-2-butanone |
|  | 254 | Methyl 4-hydroxycinnamate |
|  | 255 | Rubrofusarin-6-O-beta-D-gentiobioside |
|  | 256 | Ethyl salicylate |
| Polyphenols | 257 | kaempferide |
|  | 258 | Cimifugin beta-D-glucopyranoside |
|  | 259 | 5-O-Methylvisammioside |
| Quinones | 260 | Protohypericin |
|  | 261 | alpha-Tocopherolquinone |
|  | 262 | Embelin |
|  | 263 | Acetoxyisovalerylalkannin |
|  | 264 | Acetyl shikonin |
|  | 265 | β,β-Dimethylacrylalkannin |
|  | 266 | Alkannin |
|  | 267 | beta, beta-dimethylacrylshikonin |
|  | 268 | Lapachol |
|  | 269 | Juglone |
|  | 270 | Dimethylacrylshikonin |
|  | 271 | 2-Methoxynaphthoquinone |
|  | 272 | Rheic Acid |
|  | 273 | Lawsone |
|  | 274 | Alizarin |
|  | 275 | Aloe-emodin |
|  | 276 | Plumbagin |
|  | 277 | Ketoisophorone |
|  | 278 | Vitamin K1 |
|  | 279 | Antrapurol |
|  | 280 | Menadione |
| Steroids | 281 | Desacetylcinobufagin |
|  | 282 | Sitostenone |
|  | 283 | Taurodeoxycholate sodium salt |
|  | 284 | Sodium taurochenodeoxycholate |
|  | 285 | Fucosterol |
|  | 286 | Pennogenin 3-O-beta-chacotrioside |
|  | 287 | Qingyangshengenin |
|  | 288 | Polyphyllin VI |
|  | 289 | Tenacissoside H |
|  | 290 | Tenacissoside I |
|  | 291 | Tenacissoside G |
|  | 292 | Ruscogenin |
|  | 293 | Ophiopogonin D |
|  | 294 | Dioscin |
|  | 295 | Periplocin |
|  | 296 | Prosapogenin A |
|  | 297 | Pseudoprotodioscin |
|  | 298 | Bufotaline |
|  | 299 | Arenobufagin |
|  | 300 | Tigogenin |
|  | 301 | (25RS)-Ruscogenin |
|  | 302 | Guggulsterone E&Z |
|  | 303 | GLYCODEOXYCHOLIC ACID |
|  | 304 | Periplogenin |
|  | 305 | Hecogenin |
|  | 306 | TOMATIDINE HYDROCHLORIDE |
|  | 307 | Officinalisinin I |
|  | 308 | Liriopesides B |
|  | 309 | Polyphyllin I |
|  | 310 | Diosgenin glucoside |
|  | 311 | Sitogluside |
|  | 312 | Cyasterone |
|  | 313 | Liriope muscari baily saponins C |
|  | 314 | Gracillin |
|  | 315 | Methyl protodioscin |
|  | 316 | Cinobufagin |
|  | 317 | Timosaponin AIII |
|  | 318 | Timosaponin BII |
|  | 319 | Euphorbiasteroid |
|  | 320 | Brassinolide |
|  | 321 | Caudatin |
|  | 322 | Hyodeoxycholic acid |
|  | 323 | Beta-Sitosterol |
|  | 324 | Deoxycholic acid |
|  | 325 | Cholic Acid |
|  | 326 | Cholesteryl Acetate |
|  | 327 | 5alpha-Cholestan-3-one |
|  | 328 | Cortisone |
|  | 329 | Hydroxyecdysone |
|  | 330 | Glycocholic acid |
|  | 331 | Asiatic acid |
|  | 332 | Madecassic acid |
|  | 333 | Cortodoxone |
|  | 334 | Protodioscin |
|  | 335 | Adrenosterone |
|  | 336 | Lithocholic acid |
|  | 337 | Epiandrosterone |
|  | 338 | Bufalin |
|  | 339 | 5Beta-Pregnane-3Alpha,20alpha-Diol |
|  | 340 | Hydrocortisone |
|  | 341 | Estriol |
|  | 342 | Estradiol |
|  | 343 | Aquacrine |
|  | 344 | Dehydroepiandrosterone |
|  | 345 | Pregnenolone |
|  | 346 | Chenodeoxycholic acid |
|  | 347 | Lactulose |
|  | 348 | Progesterone |
|  | 349 | 7-Ketocholesterol |

**Additional file 1: Table S5. Comparison of clinical data of enrolled participates and patients.**

| **Variable** | **Control** | **Diabetes** | **DCM** |
| --- | --- | --- | --- |
| Age (years) | 69.9±16.2 | 68.5±17.7 | 71.0±15.5 |
| Male/female | 17/18 | 20/16 | 17/16 |
| BMI (kg/m^2^) | 23.9±3.5 | 26.1±3.4 | 24.8±4.4 |
| FBG (mM) | 5.02±0.45 | 10.7±3.1* | 10.1±4.0* |
| HbA1c | 5.45±0.39 | 10.0±2.9* | 8.1±1.9* |
| EF (%) | 64.3±2.3 | 62.3±2.5 | 40.0±9.3* |

**Note**: BMI: body mass index; FBG: fasting blood glucose; HbA1c: hemoglobin A1C; EF: ejection fraction. **P* < 0.05 versus Control. Differences between groups were assessed with ANOVA followed by Bonferroni post-hoc test.

**Additional file 1: Table S6. Primers for ChIP analysis in the present study**.

| Genes | Forward primer (5′→3′) | Reverse primer (5′→3′) |
| --- | --- | --- |
| Peiostin promoter-Smad2 | CCTGTGGGGTAGGAACTGAA | TCCAGCCTCTTCTTGACCAT |
| Peiostin promoter-Smad3 | TCAAAGGCCAGACAACAGAGT | TCACAGGTAAGAACAGGGCG |
| BCACT2 promoter-H3K27ac | CCTGGGCAGGTGACTTCTAA | TGTTCATGGTTCCCACCTCG |
| PP2CM promoter-H3K27ac | GAGTCGGACGAGCCTTTCAC | TTCCAGTGGTTTTCGGCCTT |
| Col I promoter-H3K27ac | AATTTCCCCTCTCTCTGGCC | GTAGGCTACGCTGTTCTTGC |
| α-SMA promoter-H3K27ac | AGAGCTGGCATCTTCTGAGG | TCTGCTCTGCTCTCGAAACA |

**Additional file 1: Table S7**. **Biochemical characteristics and echocardiographic data of control and diabetic mice in the presence or absence of periostin**.

| Characteristics | WT | Periostin^-/-^ | Diabetes | Diabetes+Periostin^-/-^ |
| --- | --- | --- | --- | --- |
| Body weight | 30.2±1.4 | 31.1±1.3 | 29.9±1.4 | 30.1±1.5 |
| FBG (mmol/l) | 7.61±0.72 | 7.43±0.82 | 20.01±2.47* | 21.32±2.36* |
| HbA1c (%) | 4.24±0.41 | 4.34±0.34 | 12.12±2.12* | 11.87±1.89* |
| Total cholesterol (mmol/l) | 2.61±0.22 | 2.62±0.24 | 3.81±0.32* | 3.72±0.33* |
| Triacylglycerols (mmol/l) | 1.22±0.13 | 1.24±0.12 | 2.74±0.24* | 2.71±0.26* |
| LVIDD (mm) | 3.61±0.32 | 3.67±0.33 | 4.53±0.51* | 3.74±0.42† |
| LVPWD (mm) | 1.64±0.13 | 1.65±0.14 | 0.84±0.072* | 1.32±0.15† |
| LVIDS (mm) | 2.14±0.25 | 2.22±0.23 | 3.33±0.32* | 2.32±0.23† |
| LVAPWD (mm) | 1.62±0.16 | 1.61±0.16 | 0.82±0.092* | 1.55±0.14† |

Notes: WT: wild-type; FBG, fasting blood glucose; HbA1c, Hemoglobin A1C; LVEF, left ventricular ejection fraction; LVIDD, left ventricular internal diameter in diastolic end; left ventricular internal diameter in systolic end; LVFS, left ventricular fraction shortening; LVIDS, LVPWD, left ventricular posterior wall thickness in diastolic end; LVAPWD, left ventricular anterior wall thickness in diastolic end. n =6. **P* < 0.05 versus WT, †*P* < 0.05 versus Diabetes. Differences between groups were assessed with ANOVA followed by Bonferroni post-hoc test.

**Additional file 1: Table S8**. **Biochemical characteristics and echocardiographic data of control and diabetic mice after overexpression of periostin**.

| Characteristics | Con | Periostin OE | Diabetes | Diabetes+Periostin OE |
| --- | --- | --- | --- | --- |
| Body weight | 30.1±1.5 | 31.2±1.4 | 29.5±1.6 | 31.2±1.8 |
| FBG (mmol/l) | 7.33±0.76 | 7.29±0.81 | 21.02±2.55* | 20.11±2.15* |
| HbA1c (%) | 4.21±0.42 | 4.22±0.31 | 12.11±2.17* | 11.55±2.08* |
| Total cholesterol (mmol/l) | 2.58±0.31 | 2.61±0.23 | 3.65±0.31* | 3.71±0.37* |
| Triacylglycerols (mmol/l) | 1.23±0.12 | 1.22±0.14 | 2.75±0.22* | 2.81±0.23* |
| LVIDD (mm) | 3.55±0.31 | 4.22±0.32 | 4.57±0.58* | 5.71±0.58† |
| LVPWD (mm) | 1.61±0.14 | 0.93±0.12 | 0.85±0.089* | 0.67±0.072† |
| LVIDS (mm) | 2.23±0.21 | 3.21±0.22 | 3.35±0.34* | 4.48±0.37† |
| LVAPWD (mm) | 1.61±0.13 | 0.84±0.091 | 0.83±0.095* | 0.67±0.073† |

Notes: WT: wild-type; FBG, fasting blood glucose; HbA1c, Hemoglobin A1C; LVEF, left ventricular ejection fraction; LVIDD, left ventricular internal diameter in diastolic end; left ventricular internal diameter in systolic end; LVFS, left ventricular fraction shortening; LVIDS, LVPWD, left ventricular posterior wall thickness in diastolic end; LVAPWD, left ventricular anterior wall thickness in diastolic end. n =6. **P* < 0.05 versus Con, †*P* < 0.05 versus Diabetes. Differences between groups were assessed with ANOVA followed by Bonferroni post-hoc test.

**Additional file 1: Table S9**. **Biochemical characteristics and echocardiographic data of control and diabetic mice after treatment of GA**.

| Characteristics | Con | Diabetes | Diabetes+GA  (10 mg/kg) | Diabetes+GA  (20 mg/kg) | Diabetes+GA  (40 mg/kg) |
| --- | --- | --- | --- | --- | --- |
| Body weight | 29.6±1.5 | 29.1±1.8 | 29.7±1.6 | 30.4±1.6 | 29.3±1.4 |
| FBG (mmol/l) | 7.34±0.74 | 21.04±2.44* | 20.34±2.31* | 20.82±2.44* | 21.29±2.55* |
| HbA1c (%) | 4.21±0.51 | 12.33±1.57* | 11.14±1.78* | 12.01±1.72* | 11.99±1.67* |
| Total cholesterol (mmol/l) | 2.58±0.25 | 3.82±0.36* | 3.77±0.31* | 3.79±0.35* | 3.66±0.34* |
| Triacylglycerols (mmol/l) | 1.21±0.12 | 2.81±0.23* | 2.78±0.25* | 2.74±0.24* | 2.79±0.25* |
| LVIDD (mm) | 3.54±0.31 | 4.55±0.46* | 3.88±0.34† | 3.78±0.33† | 3.72±0.35† |
| LVPWD (mm) | 1.62±0.11 | 0.79±0.086* | 1.39±0.12† | 1.36±0.13† | 1.34±0.17† |
| LVIDS (mm) | 2.11±0.21 | 3.36±0.31* | 2.39±0.25† | 2.41±0.28† | 2.31±0.29† |
| LVAPWD (mm) | 1.64±0.17 | 0.81±0.087 | 1.59±0.13† | 1.52±0.12† | 1.56±0.11† |

Notes: WT: wild-type; FBG, fasting blood glucose; HbA1c, Hemoglobin A1C; LVEF, left ventricular ejection fraction; LVIDD, left ventricular internal diameter in diastolic end; left ventricular internal diameter in systolic end; LVFS, left ventricular fraction shortening; LVIDS, LVPWD, left ventricular posterior wall thickness in diastolic end; LVAPWD, left ventricular anterior wall thickness in diastolic end. n =6. **P* < 0.05 versus Con, †*P* < 0.05 versus Diabetes. Differences between groups were assessed with ANOVA followed by Bonferroni post-hoc test.
