# Supplementary material for: Disrupted cardiac fibroblast BCAA catabolism contributes to diabetic cardiomyopathy via a periostin/NAP1L2/SIRT3 axis
Source: Cell Mol Biol Lett. 2023 Nov 22;28:93. doi: 10.1186/s11658-023-00510-4 (PMC10666354; doi:10.1186/s11658-023-00510-4)
Supplement: Supplementary file 2 — Additional file 2: Fig. S1. Effects of HG, TGF-β1 and Ang II on the mRNA level of periostin in CF. Fig. S2. Expression of target proteins after knockdown or overexpression. Fig. S3. Cardiac fibroblasts-secreted periostin induced hypertrophy and apoptosis in primary neonatal cardiomyocytes. Fig. S4. Periostin is induced by HG in a TGF-β/Smad dependent manner. Fig. S5. Binding of Smad2/3 to the promoters of periostin. Fig. S6. Periostin upregulated NAP1L2 via suppressing miR-27b-3p. Fig. S7. NAP1L2 regulated SIRT3 in CF. Fig. S8. Heatmap of mean frailty score in quartiles of serum BCAAs in control and diabetic mice. Fig. S9. Effects of BCAT2 and PP2Cm on the myofibroblast differentiation of CF. Fig. S10. H3K27acoccupancy at the promoters of α-SMA and Col I in CF transfected with NAP1L2 OE plasmid by ChIP. Fig. S11. Effects of different compounds on periostin luciferase reporter activity and expression. Fig. S12. Effects of GA on the BCAA catabolism in mouse hearts. [file 11658_2023_510_MOESM2_ESM.docx]

**Additional Material**

**Reagents and chemicals**

Streptozocin (STZ, S0130), dihydroethidium (DHE, 38483–26-0) and DAPI (D9542) were procured from Sigma (St Louis, USA). Mouse periostin ELISA kit (ab243689), human periostin ELISA kit (ab213816), Lipid Peroxidation (MDA) assay kit (ab118970) and Picro Sirius Red Stain kit (Connective Tissue Stain) (ab150681) were procured from Abcam (Cambridge, USA).  5-ethynyl-2′-deoxyuridine (EdU) Alexa Fluor™ 488 imaging kits (C10637), Click-iT™ Plus TUNEL Alexa Fluor™ 594 (C10618) and Hoechst 33342 (C10246) were procured from Thermo Fisher Scientific (Carlsbad, CA, USA). Glucosyringic acid (GA, HY-N3953), recombinant transforming growth factor β1 (TGF-β1) (HY-P70543), ADTL-SA1215 (HY-139742), LY294002 (HY-10108), MK-2206 (HY-108232), and angiotensin II (Ang II, HY-13948) were purchased from MCE (Monmouth Junction, NJ, USA). Human transforming growth factor β1 (TGF-β1, #8915) was procured from Cell Signaling Technology (Danvers, Massachusetts, USA). Control siRNA (sc-36869), Smad2 siRNA (sc-77325), Smad3 siRNA (sc-77326), BCAT2 siRNA (sc-141665), PP2Cm (sc-152141), periostin siRNA (sc-270567), BCAT2 activation plasmids (sc-419298-ACT), and PP2Cm activation plasmids (sc-436297-ACT) were purchased from Santa Cruz (Santa Cruz, CA, USA). NAP1L2 siRNA (SR515136), Smad2 overexpression plasmid (RN212493), Smad3 overexpression plasmid (RN201118), periostin overexpression plasmid (RN204952) were bought from OriGen (Rockville, MD, USA). The primers were synthesized and provided by Sangon Biotech. The primary and second antibodies were listed in **Table S1**.

**Cell transfection**

The primary myocardial fibroblasts were transfected with the negative control siRNA (100 nM), Smad2 siRNA (100 nM), Smad3 siRNA (100 nM), periostin siRNA (100 nM), BCAT2 siRNA (100 nM), PP2Cm siRNA (100 nM), NAP1L2 siRNA (100 nM), BCAT2 activation plasmids (1 μg), or PP2Cm activation plasmids (1 μg) using Lipofectamine 2000 (Invitrogen, CA, USA). In addition, the primary cardiomyocytes were transfected with empty vectors (1 μg), Smad2 overexpression plasmid (1 μg), Smad3 overexpression plasmid (1 μg), or periostin overexpression plasmid (1 μg) using Lipofectamine 2000 (Invitrogen, CA, USA). After 48 h of transfection, the cells were challenged by normal glucose or high glucose for 24 h, and were then harvested for subsequent experiments. To minimize the potential influence of the reagent or transfection protocol on the target protein, scrambled sequences of the miR-27b-3p mimic and inhibitor served as negative controls (Ruibo, Guangzhou, China). CF (approximately 5 × 10^5^ cells per well) were cultured for 18 h in 6-well plates. They were then transfected with miR-27b-3p mimics (50 nM) or inhibitors (100 nM), along with their corresponding negative controls, using the RNAifectin™ transfection reagent. After transfection for 6 h, the culture medium was refreshed, the transfection reagent was removed, and the transfection efficiency was assessed after transfection for 24 h.

**Dual-luciferase reporter assay**

CF with a confluence ranging from 85% to 90% were co-transfected with pcDNA-NAP1L2 reporter plasmids or pcDNA-NAP1L2 mutated reporter plasmids (1 µg/mL), which were provided by Generay (Shanghai, China). Subsequently, the cells were incubated with Lipofectamine™3000 transfection reagent, along with either normal control or miR-27b-3p mimics (50 nm). To assess luciferase activity, a dual-luciferase reporter assay system was employed.

**Real-time fluorescence quantitative polymerase chain reaction (RT-PCR)**

Total RNA in each sample was extracted using the TRIzol reagent (Invitrogen, CA, USA) according to the manufacturer’s instructions, followed by the cDNA synthesis with the aid of Hifair® III 1st Strand cDNA Synthesis SuperMix (Yeasen, China). In compliance with manufacturer’s protocols, the RT-PCR was carried out using Hieff® qPCR SYBR Green Master Mix (Yeasen, China). The expression levels of the target gene were relative to β-actin and their expression was relatively quantified by the 2^−ΔΔCt^ method. The prime sequences were shown in **Table** **S2-3**.

**Immunoblotting and immunoprecipitation**

The cardiac tissues and cell samples were extracted in cell lysis buffer (P0013, Beyotime, Shanghai, China) containing Tris (20mM, pH7.5), 150mM NaCl, 1% Triton X-100，and sodium pyrophosphate，β-glycerophosphate，EDTA，Na_3_VO_4_，and leupeptin, and then centrifuged at 12,000 g for 15 min at 4°C, and the supernatants in each sample were collected. Total protein in each sample was quantified using a BCA protein assay kit. The protein level was normalized and the 5× loading buffer was added, following boiled for 5 min at 100 °C. Then, the equal amount of protein was electrophoresed on SDS-PAGE and transferred to PVDF membranes. After blocking for 1 h by 5% skimmed milk, the membranes were incubated with the required primary antibodies overnight at 4 °C. The membranes were then probed with horseradish peroxidase- (HRP-) conjugated secondary antibodies for 1 h, and the blot bands were visualized using enhanced chemiluminescence (WBKLS0100, Millipore, Billerica, MA, USA). The band intensities were analyzed and normalized by the loading controls using ImageJ gel analysis software. For immunoprecipitation assays, the samples were lysed with 0.5 mL of cell lysis buffer, and then centrifuged for 15 min to obtain the supernatants. The supernatants were immunoprecipitated by indicated antibodies for 2 h at 4 °C, and the Protein G PLUS-Agarose was then added on a rocker platform. The precipitates were washed 3 times with lysate, and the pellets were then resuspended in electrophoresis sample buffer (40 μl) and boiled for 5 min, and the immune complexes were subjected to immunoblotting.

**Mouse cytokine antibody array**

A commercial mouse cytokine antibody array containing 40 cytokines was used to detect the levels of cytokines in the heart tissues from control mouse and diabetic mice. A total of 125 microgram protein in each sample was used for the detection of cytokines by RayBio® C-Series Mouse Cytokine Antibody Array 7 in accordance with the manufacturer's protocol (RayBiotech, Inc., GA, USA). The results were quantified by RayBio Q Analyzer software (RayBiotech, Inc., GA, USA).

**Enzyme-linked immunosorbent assay**

The protein levels of periostin in the culture supernatants, and human serum samples, as well as mouse heart and blood samples were measured by periostin ELISA kits (R&D Systems, Minneapolis, MN, USA) following the manufacturer’s instructions as previously described [[1](#_ENREF_1)].

**Serum collection**

The serum samples in mice were obtained through centrifugation for 20 min at 3000 rpm and then stored at -80 °C. The serum levels of lactate dehydrogenase (LDH) and creatine kinase-MB (CK-MB) were analyzed by Hitachi Automatic Biochemical Analyzer (Hitachi Co., Ltd., Japan) as previously described [[2](#_ENREF_2)].

**Detection of glycolipid metabolism indicators**

The fasting blood glucose (FBG) levels of all mice were detected and recorded. Moreover, serum levels of triglycerides (TG), total cholesterol (TC), and low-density lipoprotein cholesterol (LDL-C) were enzymatically analyzed using commercial kits (Nanjing Jiancheng Bioengineering Institue, Nanjing, China) in compliance with the manufacturer’s instructions as we previously reported [[3](#_ENREF_3), [4](#_ENREF_4)].

**Measurement of oxidative stress**

The sectioned hearts and fixed CF were stained with the DHE fluorescent probe (10 μM) for an hour at 37 °C under the dark environment. After washing with PBS 3 times, the red immunofluorescence was photographed by a fluorescence microscope (80i, Nikon, Tokyo, Japan). The data were analyzed by the IMAGE-PRO PLUS 6.0 (Version 6.0, Media Cybernetics, Bethesda, Maryland, USA). In addition, the contents of MDA in each sample were detected by commercial kits based on the reaction of MDA and thiobarbituric acid (TBA) to produce a red product which is quantitatively examined by a colorimetric method at the absorbance of 532 nm. The results of MDA were normalized to the protein content in each sample.

**Plasmid constructs**

To determine the association of SIRTs with NAP1L2 sequencing encoding full-length of SIRT1-7 were cloned into pcDNA5-Flag vectors to yield pcDNA5-Flag-SIRT1, pcDNA5-Flag-SIRT2, pcDNA5-Flag-SIRT3, pcDNA5-Flag-SIRT4, pcDNA5-Flag-SIRT5, pcDNA5-Flag-SIRT6, and pcDNA5-Flag-SIRT7. These plasmids were transfected to HEK293 cells, and their interactions were examined by immunoblotting.

**Measurement of branched-chain amino acids (BCAAs)**

Serum and cardiac BCAA levels were determined by using commercial ELISA kits (Abcam, Cambridge, USA) following the manufacturer’s standards and protocols. The contents of BCAAs in the hearts were normalized to the protein levels in each sample.

**Immunofluorescence**

The cardiac tissues were cut into 5-μm sections and then dewaxed in xylene 3 times, and hydrated in alcohol. The sections were rinsed with PBS buffer and distilled water, and subjected to heat-repaired antigen for 10 min. After blocking with 1% BSA and 0.2% Triton-X for 5 min, the sections were incubated with periostin anti-rabbit antibody (1:200, Abcam) at 4 °C overnight. Next, the sections were probed by Goat anti-Rabbit IgG connected with Alexa Fluor-488 for 1 h at room temperature, followed by treatment with DAPI staining solution for 10 min. The immunofluorescence signals were captured by a fluorescence microscope (80i, Nikon, Tokyo, Japan). For cells, the fixed cardiomyocytes were incubated with anti-a-actinin antibody was added and incubated at 4 °C overnight, followed by incubation of Goat anti-Mouse IgG H&L Alexa Fluor-594 for 1 h at room temperature. The results were obtained by a fluorescence microscope (80i, Nikon, Tokyo, Japan).

**Luciferase reporter assay**

The periostin promoter constructs containing serial deletions were constructed to depict the potential regions on periostin promoters wherein periostin granted its actions in cardiomyocytes in the context of hyperglycemia. The full-length promoter region of the periostin gene from −2995 bp to the transcription start site, and a series of periostin promoters were amplified by PCR and cloned into the pGL3 luciferase vector (Promega). The periostin promoter luciferase vectors and its deletion mutants were transfected to cardiomyocytes using lipofectamine 2000 transfection reagent (Invitrogen) in the presence or absence of hyperglycemia. The firefly relative luciferase activity was measured by a dual luciferase reported gene assay kit (Promega).

**Molecular docking**

The structure of periostin (5YJH) was downloaded from the Protein Data Bank database ([https://www.rcsb.org](https://www.rcsb.org/)) according to UniProt ID, and the structure of GA was downloaded from the PubChem database (<https://pubchem.ncbi.nlm.nih.gov/>). The target protein receptor molecules were subjected to hydrogenation and charge calculation by AutoDockTools (version 4.2.6) with the binding site of the protein’s own ligand as the active pocket. Molecular docking was assessed using Vina 1.5.6. Finally, the 3D map of the molecular docking results was displayed with PyMOL software.

**Surface plasmon resonance (SPR) assay**

The SPR assays were performed to determine the binding affinity of GA with the periostin protein (Proteintech, Rosemont, IL, USA) using a Biacore T200 machine with sensor Chip CM5 (GE Healthcare, United States) at 25°C. Human recombinant periostin protein was captured on a CM5 chip via a standard amine coupling procedure, and the sensorgrams were recorded by injecting various concentrations of GA over the immobilized proteins surface. Eventually, the equilibrium dissociation constant (KD) value was calculated with the aid of the Biacore T200 evaluation software (GE Healthcare, United States).

**Drug screening**

A small molecule pool containing 349 natural products was selected to determine their effects on the periostin promoter activities using a luciferase reporter gene assay ([**Table** **S4**](https://www.ncbi.nlm.nih.gov/pmc/articles/PMC9414852/table/molecules-27-05336-t002/)). In brief, the full-length promoter region of the periostin gene from −2995 bp to the transcription start site cloned into the pGL3 luciferase vector (Promega) and the periostin luciferase reporter gene plasmids were transfected with cardiomyocytes for 48 h, and different compounds (5 μM) were added in the presence of high glucose for 24 h. The firefly relative luciferase activity was measured by a dual luciferase reported gene assay kit (Promega).

**Determination of BCAAs concentrations using targeted metabolomics**

A targeted metabolomics strategy was applied for the measurement of serum BCAAs concentrations as previously described [[5-7](#_ENREF_5)]. In short, a total of 0.05 mL portions of calibrators or serum samples were combined with an equal volume of the isotopically labeled internal standard solution. The amino acids were then extracted using 0.4 mL of acetonitrile containing 0.1% formic acid and subsequently analyzed via isotope dilution liquid chromatography tandem mass spectrometry (LC/MS/MS) with positive electronic spray ionization in the multiple reaction monitoring mode. Numerically, BCAAs means the sum of the concentrations of serum [leucine](https://www.sciencedirect.com/topics/medicine-and-dentistry/leucine) (Leu), [isoleucine](https://www.sciencedirect.com/topics/medicine-and-dentistry/isoleucine) (Ile), and [valine](https://www.sciencedirect.com/topics/medicine-and-dentistry/valine) (Val).

**RNA sequencing**

The transcriptome sequencing and analysis were conducted by OE Biotech Co., Ltd. (Shanghai, China). In short, total RNA was isolated using the TRIzol reagent (Invitrogen, CA, USA) according to the manufacturer’s protocol. RNA purity and quantification were evaluated using the NanoDrop 2000 spectrophotometer (Thermo Scientific, USA). RNA integrity was assessed using the Agilent 2100 Bioanalyzer (Agilent Technologies, Santa Clara, CA, USA). The libraries were constructed using VAHTS Universal V6 RNA-seq Library Prep Kit according to the manufacturer’s instructions. The libraries were sequenced on a llumina Novaseq 6000 platform and 150 bp paired-end reads were generated. Raw reads of fastq format were firstly processed using fastp1 and the low quality reads were removed to obtain the clean reads. The clean reads were mapped to the reference genome using HISAT22. PCA analysis was performed using R (v 3.2.0) to evaluate the biological duplication of samples. Differential expression analysis was performed using the DESeq25. Q value < 0.05 and fold change > 2 or fold change < 0.5 was set as the threshold for significantly differential expression gene (DEGs). Hierarchical cluster analysis of DEGs was performed using R (v 3.2.0) to demonstrate the expression pattern of genes in different groups and samples. Based on the hypergeometric distribution, GO, KEGG pathway, Reactome and WikiPathways enrichment analysis of DEGs were performed to screen the significant enriched term using R (v 3.2.0), respectively. Gene Set Enrichment Analysis (GSEA) was performed using GSEA software. RNA sequencing raw data had been submitted to Sequencing Read Archieve (SRA) under accession no. PRJNA950964.


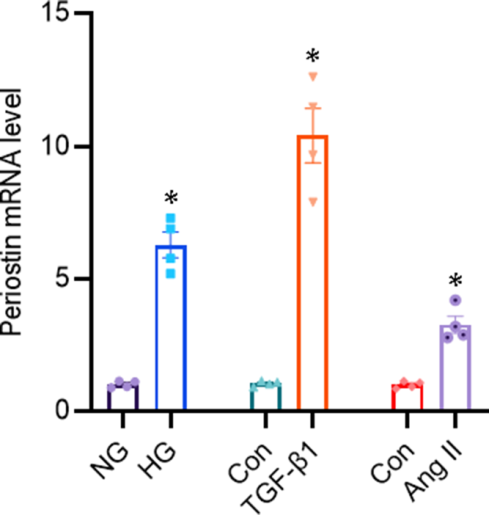


**Additional file 2: Fig. S1. Effects of HG, TGF-β1 and Ang II on the mRNA level of periostin in CF.** Primary CF were incubated with HG (33.3 mM), TGF-β1 (5 ng/mL), or Ang II (1 μg/ml) for 24 h. The mRNA level of periostin was determined by RT-PCR. n =4. **P* < 0.05 versus NG or Con. The P-value was calculated by unpaired two-tailed Student’s t-test.


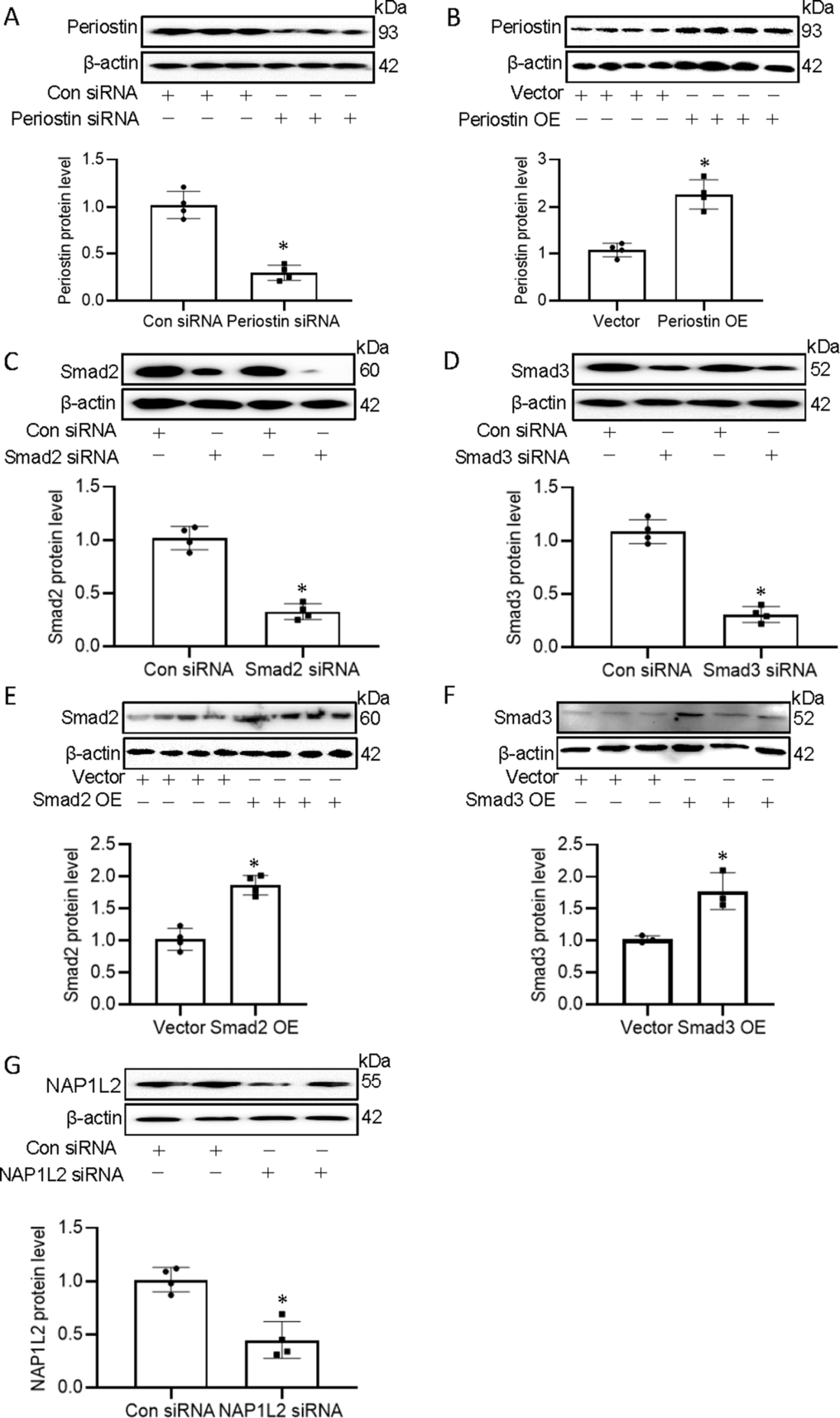


**Additional file 2: Fig. S2. Expression of target proteins after knockdown or overexpression.** (**A**) Representative blots and quantitation of periostin protein in cardiac fibroblasts after periostin overexpression (OE). (**B**) Representative blots and quantitation of periostin protein in cardiac fibroblasts after periostin siRNA transfection. (**C**) Representative blots and quantitation of Smad2 protein in cardiac fibroblasts after Smad2 siRNA transfection. (**D**) Representative blots and quantitation of Smad3 protein in cardiac fibroblasts after Smad3 siRNA transfection. (**E**) Representative blots and quantitation of Smad2 protein in cardiac fibroblasts after Smad2 overexpression (OE). (**F**) Representative blots and quantitation of Smad3 protein in cardiac fibroblasts after Smad3 overexpression (OE). (**G**) Representative blots and quantitation of NAP1L2 protein in cardiac fibroblasts after NAP1L2 siRNA transfection. n =3-4. **P* < 0.05 versus Vector or control (con) siRNA. The P-value was calculated by unpaired two-tailed Student’s t-test.


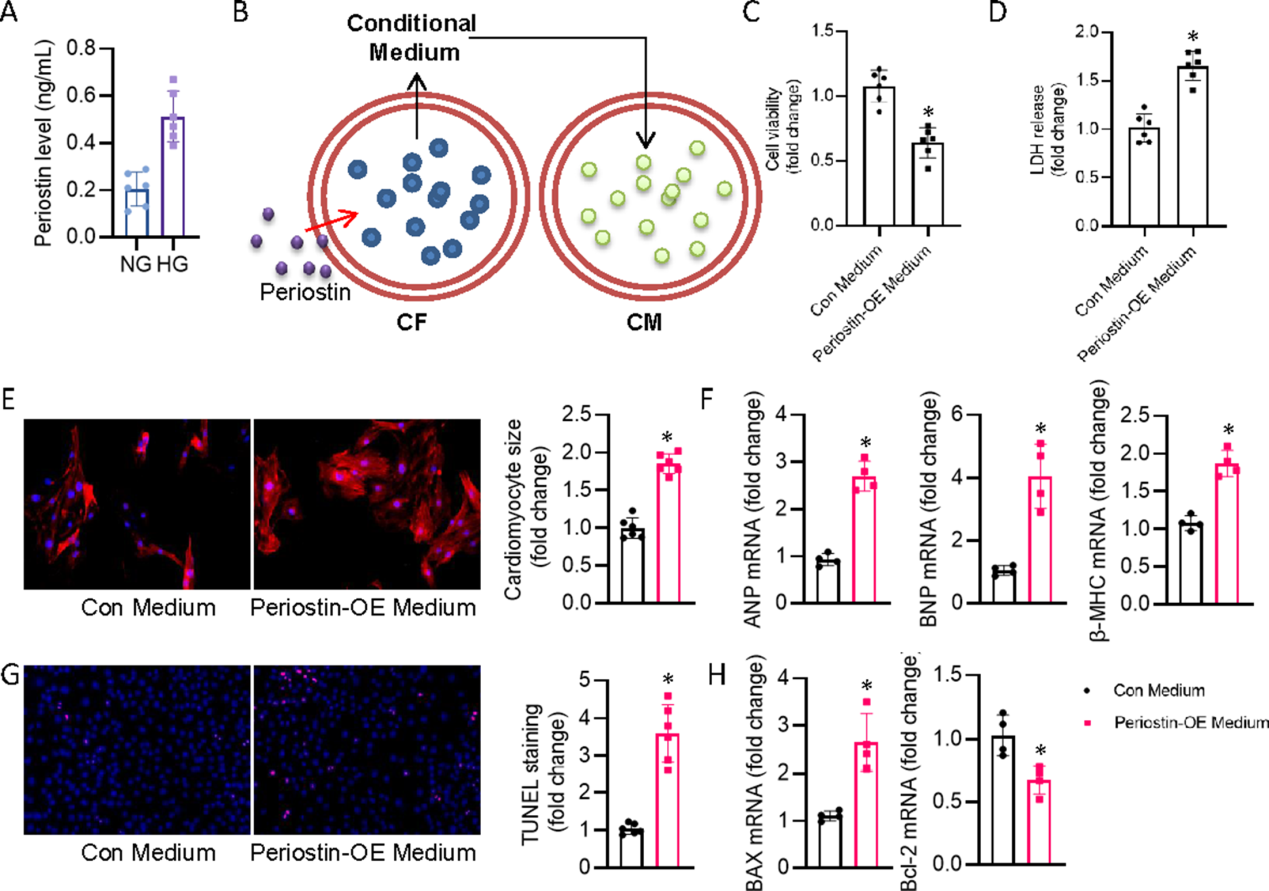


**Additional file 2: Fig. S3. Cardiac fibroblasts-secreted periostin induced hypertrophy and apoptosis in primary neonatal cardiomyocytes.** (**A**) Periostin levels in cardiac fibroblasts challenged by normal glucose (NG) or high glucose (HG). (**B**) A diagram showing the effects of conditional medium from cardiac fibroblasts with periostin overexpression (OE) on the behaviors of cardiomyocytes. (**C**) Cell viability. (**D**) LDH release. (**E**) Immunofluorescence of α-actinin. (**F**) Relative mRNA levels of *ANP*, *BNP*, and *β-MHC*. (**G**) Representative images and quantitation of TUNEL-positive cardiomyocytes. (**H**) Relative mRNA levels of *BAX* and *Bcl-2*. n =4-6. **P* < 0.05 versus NG or control (con) medium. The P-value was calculated by unpaired two-tailed Student’s t-test.


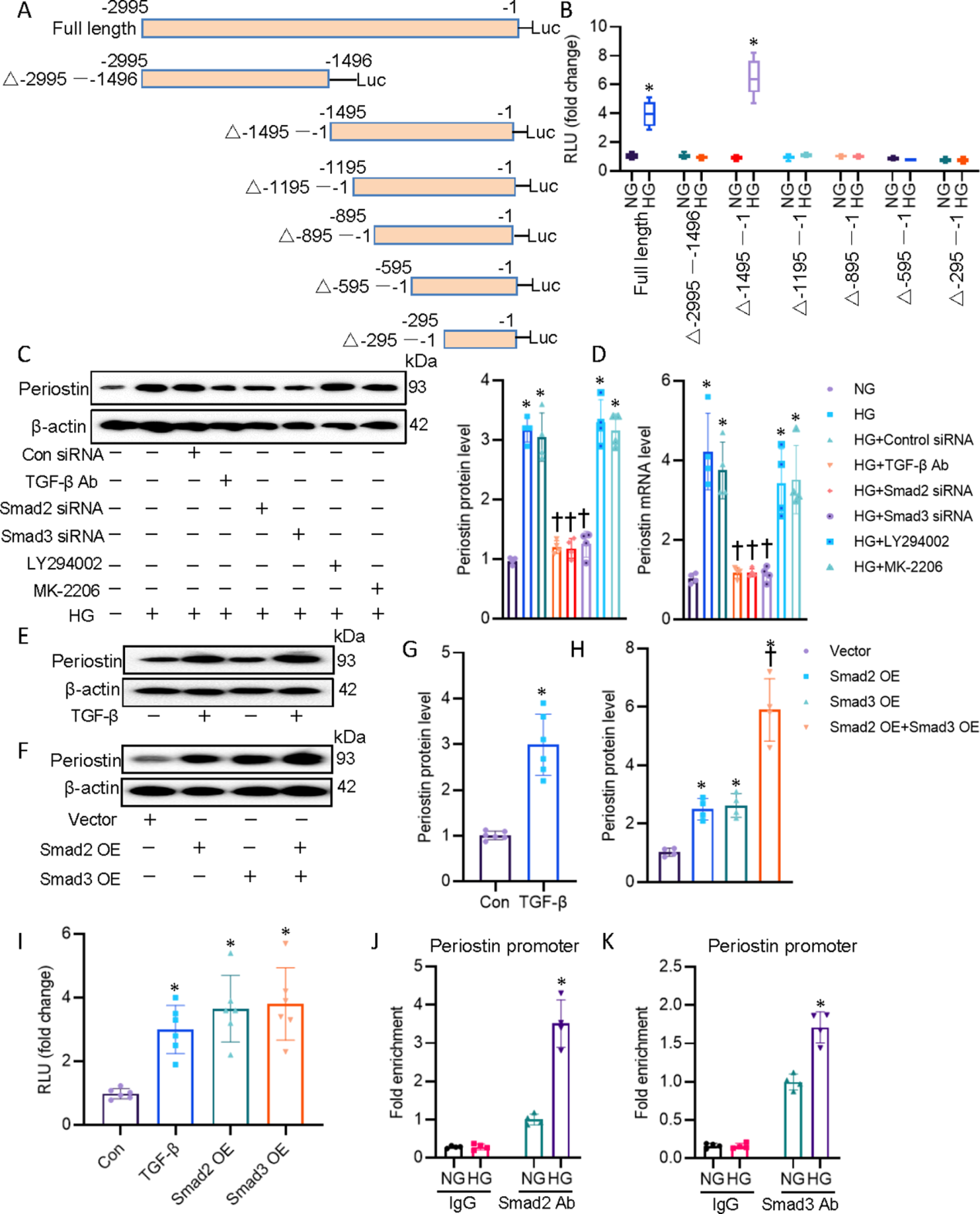


**Additional file 2: Fig. S4**. **Periostin is induced by HG in a TGF-β/Smad dependent manner.** (**A, B**) Relative luciferase activity derived from series of deletion mutants of periostin promoter constructs in CF. (**C**) Effects of blockaded of TGF-β/Smad or PI3K/Akt pathway on the protein expression of periostin in HG-exposed CF. (**D**) Effects of blockaded of TGF-β/Smad or PI3K/Akt pathway on the mRNA level of periostin in HG-exposed CF. (**E, G**) Representative blots and quantitation of periostin protein in CF treated with TGF-β1. (**F, H**) Representative blots and quantitation of periostin protein in CF with Smad2/3 overexpression (OE). (**I**) The effects of TGF-β/Smad on the relative luciferase activity of periostin promoters. (**J**) Relative quantitation of precipitated DNA determined with chromatin immunoprecipitation analysis using Smad2 antibody. (**K**) Relative quantitation of precipitated DNA determined with chromatin immunoprecipitation analysis using Smad2 antibody. n =4-6. **P* < 0.05 versus NG or control (con), †*P* < 0.05 versus HG. The P-value was calculated by unpaired two-tailed Student’s t-test (B, J, G, K). Differences between groups were assessed with ANOVA followed by Bonferroni post-hoc test (C, F, I).


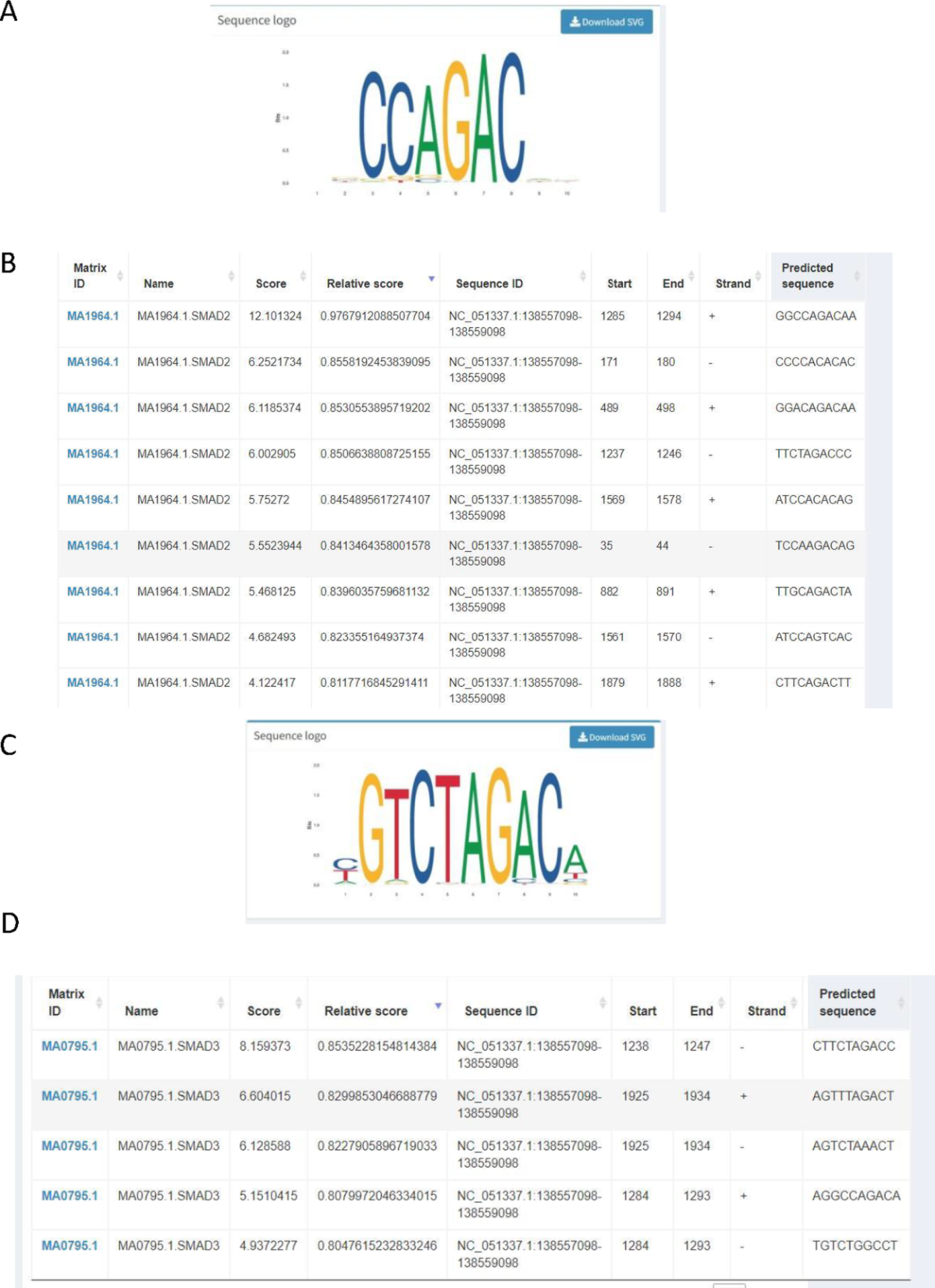


**Additional file 2: Fig. S5. Binding of Smad2/3 to the promoters of periostin.** (**A**) Consensus DNA-binding motifs of Smad2 according to JASPAR database. (**B**) Predicting the binding sites of Smad2 within periostin promoters. (**C**) Consensus DNA-binding motifs of Smad3 according to JASPAR database. (**D**) Predicting the binding sites of Smad3 within periostin promoters.


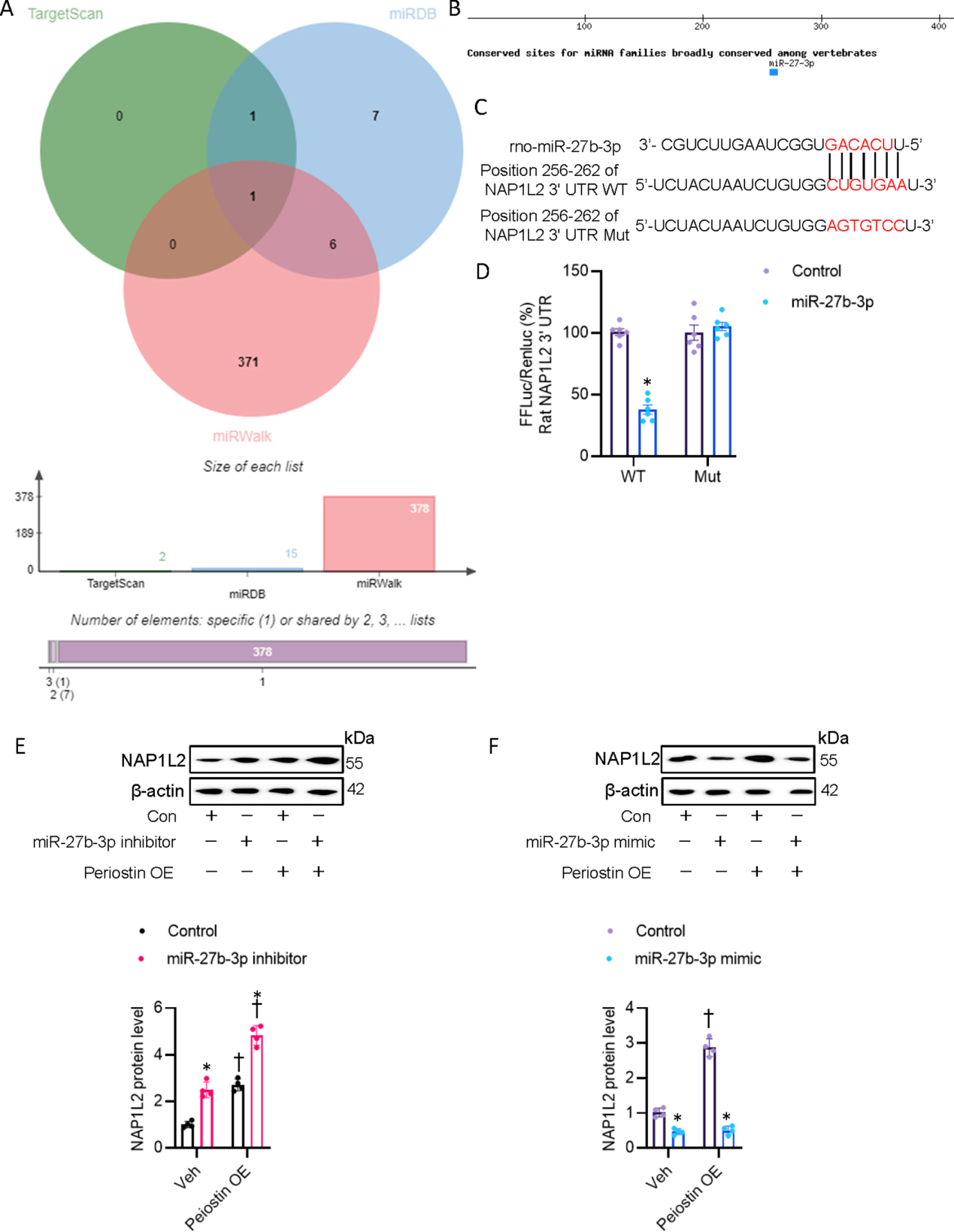


**Additional file 2: Fig. S6. Periostin upregulated NAP1L2 via suppressing miR-27b-3p.** (**A**) Heatmap showing the predicted miRNAs that regulated NAP1L2 using TargetScan, miRWalk, and miRDB databases. (**B**) Conserved sites for miRNA family for NAP1L2. (**C**)  Prediction of the location of the miR-27b-3p combination to NAP1L2 promoters by TargetScanHuman. (**D**) Dual-luciferase reporter assay showing that NAP1L2 was a target of miR-27b-3p in CF. FFLuc is firefly luciferase; Renluc is Renilla luciferase. (**E**) Effects of miR-27b-3p inhibitors on the NAP1L2 protein expression induced by periostin overexpression. (**F**) Effects of miR-27b-3p mimics on the NAP1L2 protein expression induced by periostin overexpression. n =3-4. **P* < 0.05 versus Control or Control (Con). †*P* < 0.05 versus Veh. Differences between groups were assessed with ANOVA followed by Bonferroni post-hoc test.

**
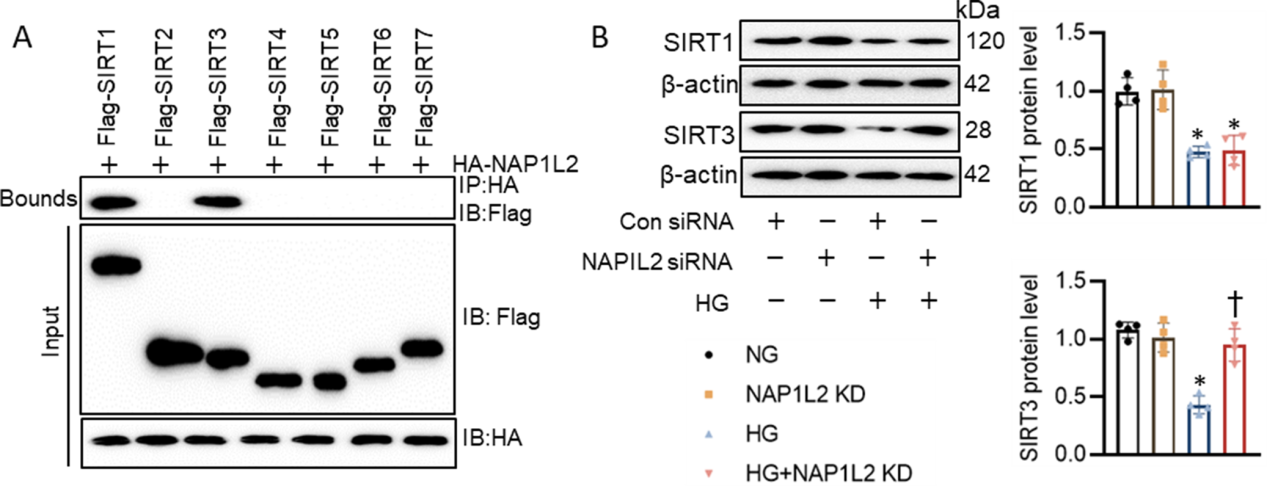
**

**Additional file 2: Fig. S7**. **NAP1L2 regulated SIRT3 in CF.** (**A**) Co-IP assays showing the potential binding between NAP1L2 and SIRT1-7. (**B**) Effects of NAP1L2 siRNA on the protein expression of SIRT1 and SIRT3. n =3-4. *P < 0.05 versus NG, †*P* < 0.05 versus HG. Differences between groups were assessed with ANOVA followed by Bonferroni post-hoc test.


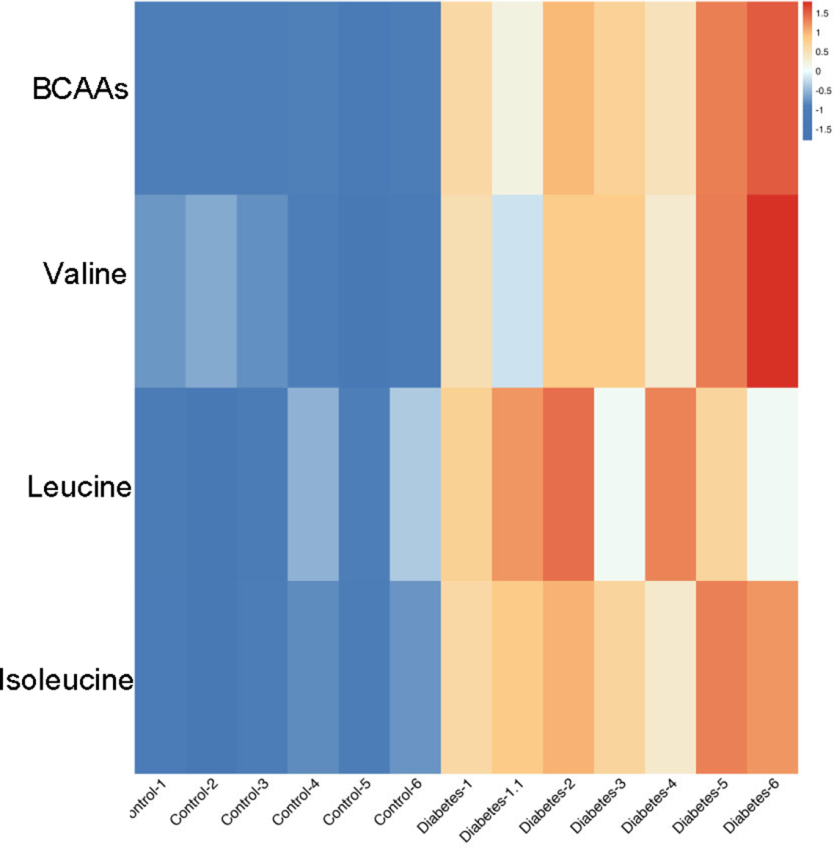


**Additional file 2: Fig. S8**. **Heatmap of mean frailty score in quartiles of serum BCAAs in control and diabetic mice.** BCAAs, branched-chain amino acids.


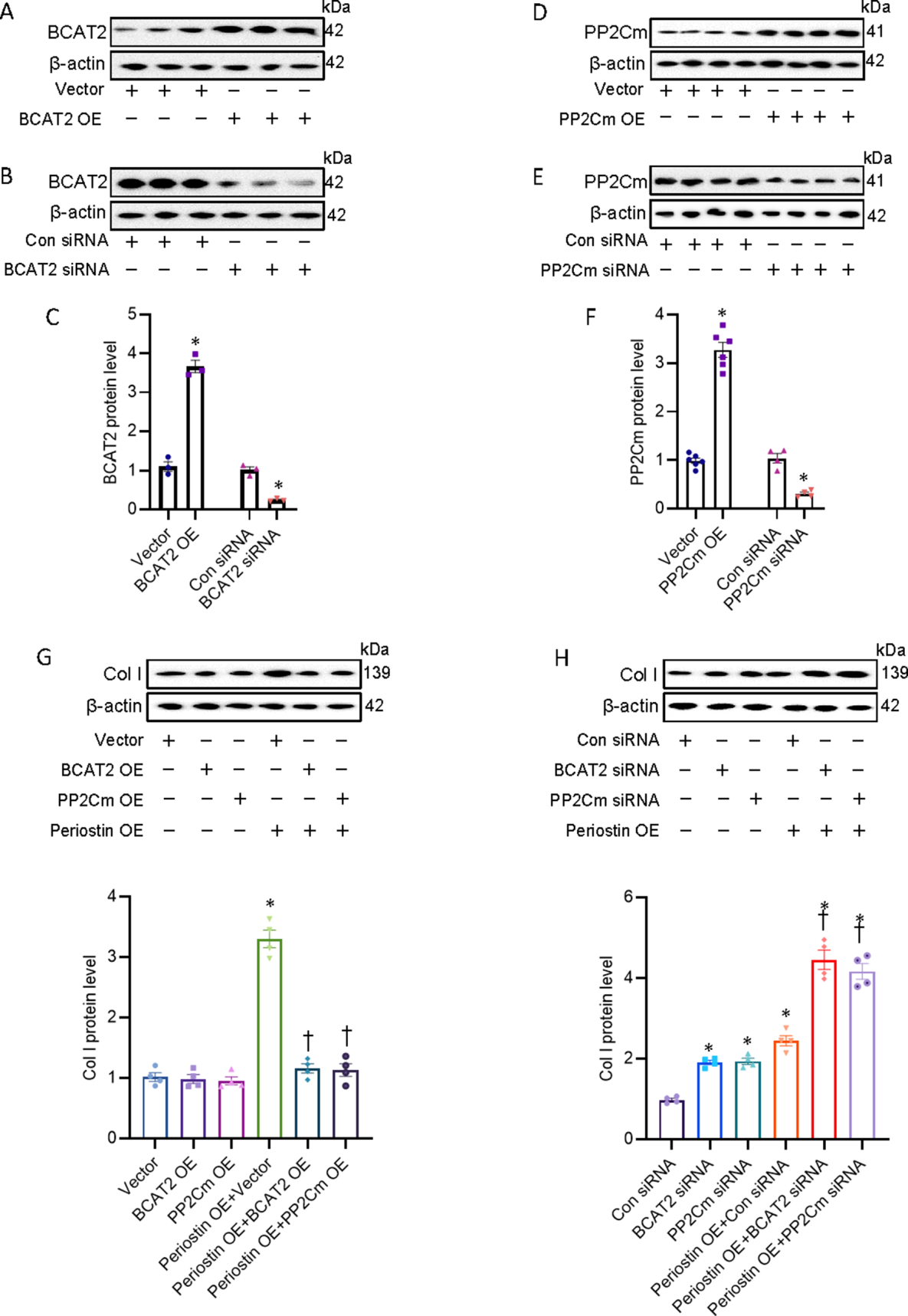


**Additional file 2: Fig. S9. Effects of BCAT2 and PP2Cm on the myofibroblast differentiation of CF.** (**A-C**) Representative blots showing the detection of BCAT2 overexpression and knockdown efficiency. (**D-F**) Representative blots showing the detection of PP2Cm overexpression and knockdown efficiency. (**G**) Effects of BCAT2 and PP2Cm overexpression on the protein expression of Col I in CF with periostin overexpression. (**G**) Effects of BCAT2 and PP2Cm downregulation on the protein expression of Col I in CF with periostin overexpression. n =4-6. **P* < 0.05 versus Vector or Control siRNA. †*P* < 0.05 versus Periostin OE+Vector or Periostin OE+Con siRNA. The P-value was calculated by unpaired two-tailed Student’s t-test (C, F). Differences between groups were assessed with ANOVA followed by Bonferroni post-hoc test (G, H).

**
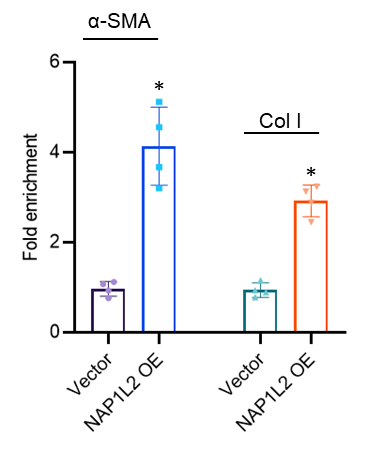
**

**Additional file 2: Fig. S10. H3K27acoccupancy at the promoters of α-SMA and Col I in CF transfected with NAP1L2 OE plasmid by ChIP.** n =4. **P* < 0.05 versus Vector. The P-value was calculated by unpaired two-tailed Student’s t-test.

**
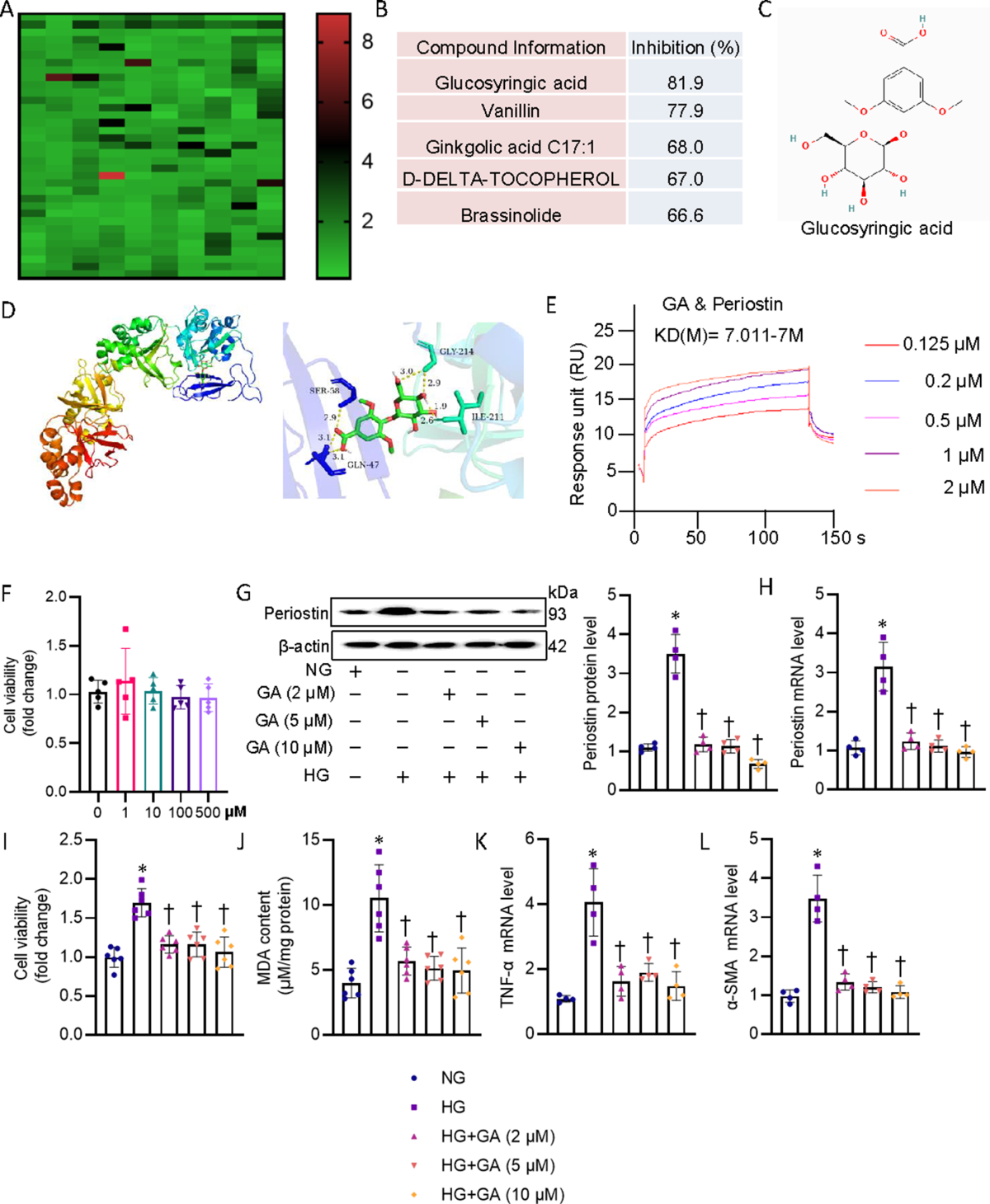
**

**Additional file 2: Fig. S11. Effects of different compounds on periostin luciferase reporter activity and expression.** (**A**) Cardiac fibroblasts were transfected with periostin luciferase reporter gene plasmid (0.5 μg) for 24 h, and then treated with 349 compounds (10 μM) for 24 h. Cell lysates were subjected to luciferase reporter assay. Heatmap showing the effects of various compounds on the periostin luciferase reporter gene activity. (**B**) Top five compounds that inhibited periostin luciferase reporter gene activity were shown. (**C**) The chemical structure of GA. (**D**) Molecular docking of GA to periostin. (**E**) SPR showing the interaction of GA with periostin. (**F**) Effects of different doses of GA on the viability of cardiac fibroblasts. (**G**) Effects of different doses of GA on the protein expression of periostin. (**H**) Effects of different doses of GA on the mRNA level of periostin. (**I**) Cell viability. (**J**) LDH release. (**K**) Relative mRNA level of TNF-α. (**L**) Relative mRNA level of α-SMA. n =4-6. **P* < 0.05 versus NG. †*P* < 0.05 versus HG. Differences between groups were assessed with ANOVA followed by Bonferroni post-hoc test.


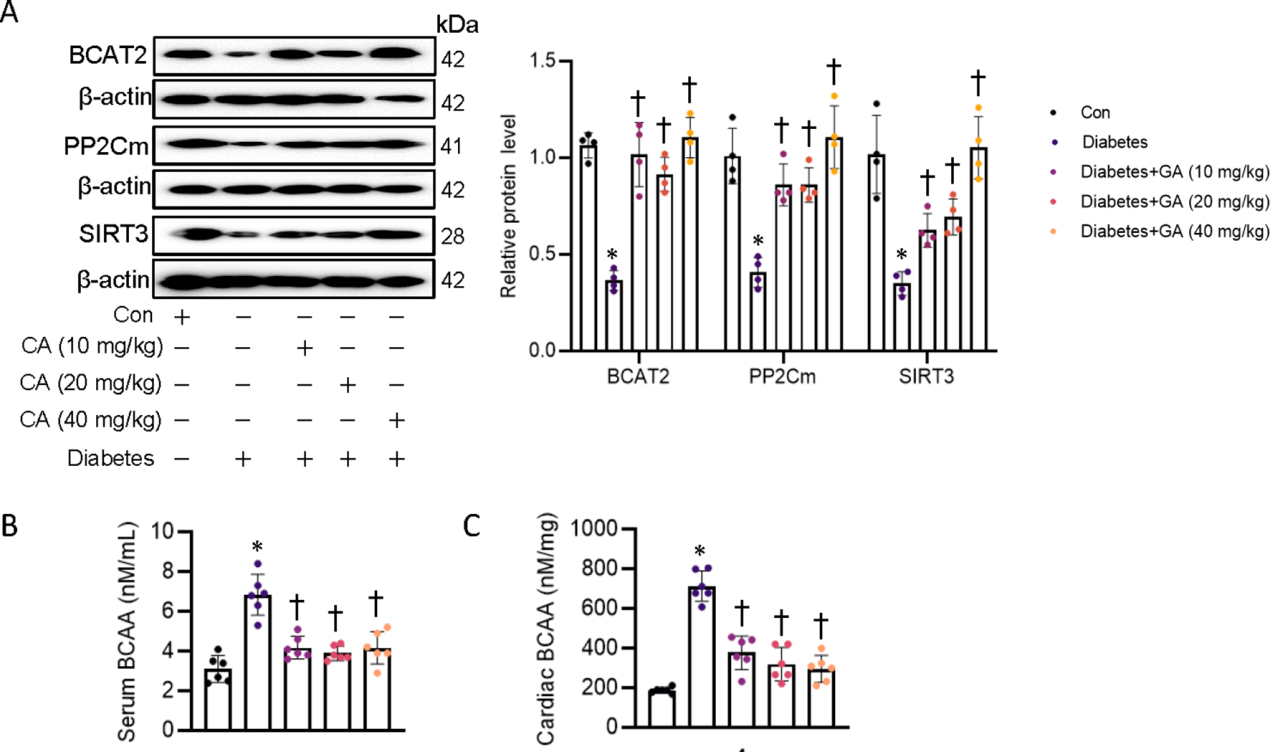


**Additional file 2: Fig. S12. Effects of GA on the BCAA catabolism in mouse hearts.** (**A**) Representative blots and quantitation of BCAT2, PP2Cm, SIRT3 in control mice, diabetic mice, and diabetic mice treated with GA. (**B**) Serum BCAA levels in control mice, diabetic mice, and diabetic mice treated with GA. (**C**) Cardiac BCAA levels in control mice, diabetic mice, and diabetic mice treated with GA. n =4-6. **P* < 0.05 versus Control (Con). †*P* < 0.05 versus Diabetes. Differences between groups were assessed with ANOVA followed by Bonferroni post-hoc test.

**References**

1. Choi S, Jung MA, Hwang YH, Pyun BJ, Lee JY, Jung DH, et al. Anti-allergic effects of Asarum heterotropoides on an ovalbumin-induced allergic rhinitis murine model. Biomedicine & pharmacotherapy = Biomedecine & pharmacotherapie. 2021; 141: 111944.

2. Ge F, Gao X, Zhou X, Li J, Ma X, Huang M, et al. The alkaloids of Corydalis hendersonii Hemsl. contribute to the cardioprotective effect against ischemic injury in mice by attenuating cardiomyocyte apoptosis via p38 MAPK signaling pathway. 2023; 18: 29.

3. Lu QB, Ding Y, Liu Y, Wang ZC, Wu YJ, Niu KM, et al. Metrnl ameliorates diabetic cardiomyopathy via inactivation of cGAS/STING signaling dependent on LKB1/AMPK/ULK1-mediated autophagy. Journal of advanced research. 2022.

4. Wang ZC, Niu KM, Wu YJ, Du KR, Qi LW. A dual Keap1 and p47(phox) inhibitor Ginsenoside Rb1 ameliorates high glucose/ox-LDL-induced endothelial cell injury and atherosclerosis. 2022; 13: 824.

5. Zhang Y, Zhou Q, Yang R, Hu C, Huang Z, Zheng C, et al. Serum branched-chain amino acids are associated with leukocyte telomere length and frailty based on residents from Guangxi longevity county. Scientific reports. 2020; 10: 10252.

6. Yang RY, Wang SM, Sun L, Liu JM, Li HX, Sui XF, et al. Association of branched-chain amino acids with coronary artery disease: A matched-pair case-control study. Nutrition, metabolism, and cardiovascular diseases : NMCD. 2015; 25: 937-42.

7. Yang R, Dong J, Guo H, Li H, Wang S, Zhao H, et al. Rapid and precise measurement of serum branched-chain and aromatic amino acids by isotope dilution liquid chromatography tandem mass spectrometry. PloS one. 2013; 8: e81144.
